# Evidence for a Common Toolbox Based on Necrotrophy in a Fungal Lineage Spanning Necrotrophs, Biotrophs, Endophytes, Host Generalists and Specialists

**DOI:** 10.1371/journal.pone.0029943

**Published:** 2012-01-11

**Authors:** Marion Andrew, Reeta Barua, Steven M. Short, Linda M. Kohn

**Affiliations:** Department of Ecology and Evolutionary Biology, University of Toronto, Mississauga, Ontario, Canada; University of California Riverside, United States of America

## Abstract

The Sclerotiniaceae (Ascomycotina, Leotiomycetes) is a relatively recently evolved lineage of necrotrophic host generalists, and necrotrophic or biotrophic host specialists, some latent or symptomless. We hypothesized that they inherited a basic toolbox of genes for plant symbiosis from their common ancestor. Maintenance and evolutionary diversification of symbiosis could require selection on toolbox genes or on timing and magnitude of gene expression. The genes studied were chosen because their products have been previously investigated as pathogenicity factors in the Sclerotiniaceae. They encode proteins associated with cell wall degradation: acid protease 1 (*acp1*), aspartyl protease (*asps*), and polygalacturonases (*pg1, pg3, pg5, pg6*), and the oxalic acid (OA) pathway: a zinc finger transcription factor (*pac1*), and oxaloacetate acetylhydrolase (*oah*), catalyst in OA production, essential for full symptom production in *Sclerotinia sclerotiorum*. Site-specific likelihood analyses provided evidence for purifying selection in all 8 pathogenicity-related genes. Consistent with an evolutionary arms race model, positive selection was detected in 5 of 8 genes. Only generalists produced large, proliferating disease lesions on excised *Arabidopsis thaliana* leaves and oxalic acid by 72 hours *in vitro*. *In planta* expression of *oah* was 10–300 times greater among the necrotrophic host generalists than necrotrophic and biotrophic host specialists; *pac1* was not differentially expressed. Ability to amplify 6/8 pathogenicity related genes and produce oxalic acid in all genera are consistent with the common toolbox hypothesis for this gene sample. That our data did not distinguish biotrophs from necrotrophs is consistent with 1) a common toolbox based on necrotrophy and 2) the most conservative interpretation of the 3-locus housekeeping gene phylogeny – a baseline of necrotrophy from which forms of biotrophy emerged at least twice. Early *oah* overexpression likely expands the host range of necrotrophic generalists in the Sclerotiniaceae, while specialists and biotrophs deploy *oah*, or other as-yet-unknown toolbox genes, differently.

## Introduction

When we consider trophic choice in microbes, we often assume rigid categories predicated on easily classified tracks of interaction with the substrate or host. The reality is more complex and more interesting. Within a single evolutionary lineage we can observe species on different trophic tracks – with evidence that shifts to biotrophy, necrotrophy and apparently symptomless endophytism can arise with apparent randomness [1]. Here we focus on the alternatives of biotrophy or necrotrophy, and of generalist or specialist host range among closely-related species within a fungal evolutionary lineage.

For purposes of this study we define terms as follows. Saprotrophs derive energy from non-living, usually inorganic material, biotrophs from living cells, necrotrophs from killed cells, and the subsidiary group, hemibiotrophs, derive nutrition initially from living cells then subsequently from killed cells [2], [3]. Endophytes occupy hosts without causing symptoms and may or may not ever cause symptoms in the host; the term endophytism is not synonymous with hemibiotrophy [4]. Host generalists have a wide host range, here spanning plant families. Specialists have a narrower host range, here mainly limited to one or few host species in one family.

The respective programs for biotrophic or necrotrophic fungal interactions with plants are increasingly well characterized by fundamental differences. Although both initially associate with a living plant, biotrophs evade or suppress the plant immune system to obtain nutrients in association with living plant tissue [5]. Recognition of biotrophic infection activates defense responses regulated by the salicylic acid (SA) signaling pathway, leading to host programmed cell death (PCD) at the site of infection, thus limiting fungal colonization [5], [6]. That some necrotrophs, requiring non-living plant tissue as a nutrient source, can actually be facilitated by host detection and response is evidenced by a number of necrotrophic species that induce host PCD, including *Botrytis cinerea* and *Sclerotinia sclerotiorum* [7]–[9]. Other necrotrophs engage or evade plant defenses, for example by detoxification of plant defense products, such as phytoalexins [10], [11]. In many interactions with necrotrophs (and herbivores), the plant successfully defends itself via jasmonic acid (JA) and ethylene-dependent pathways [5], [6].

Why is one species a necrotroph or a biotroph, a generalist or a specialist? Can we infer from analyses of primary DNA or protein sequence the evolutionary trajectories from one state to another, the type and strength of selection or the mechanisms of interaction with the host? Alternatively, do closely related species share essentially the same toolbox of pathogenesis-associated genes, but differ in timing or magnitude of gene expression? If so, are such differences in expression constitutive or induced? As a closely related species group in a monophyletic lineage, the Sclerotiniaceae of the Ascomycota could be an ideal laboratory for addressing these questions. This family has evolved relatively recently (approximately 200 million years ago) and likely radiated with the divergence of flowering plants [12], [13]. The Sclerotiniaceae includes the well-studied and economically important agricultural pathogens, host generalists, and exemplars of necrotrophy, *S. sclerotiorum* and *B. cinerea*. It also includes many species that are known not from close observation of their life histories, but instead only from the association of their sexual fruiting bodies, the phase most readily visible and most frequently collected, with one or more, wild host plant species. These include what we conclude, based on the inability to grow it on a range of synthetic media, is an obligate biotroph of poplar, *Ciborinia whetzelii* [14] and a group of facultatively biotrophic, apparently symptomlessly endophytic, host specialists on the monocot families, Juncaceae (rushes), Cyperaceae (sedges) and Poaceae (grasses) in the genus *Myriosclerotinia* [15]. Species in the section Junctoriae of *Monilinia* are necrotrophic, brown rot pathogens of wild and domesticated Rosaceae species with a random, shot-gun infection strategy mediated by asexual spores that germinate in wounds, while members of the Disjunctoriae are specialized pathogens utilizing both asexual and sexual reproduction, and attraction of insect vectors via production of sweet fragrances or sugary secretions, to infect dry stone fruits of the Rosaceae, or dry capsular fruits or sweet berry fruits of the Ericaceae [16]. In comparisons of host and fungus phylogenies, *Monilinia* species show a general pattern of co-evolution with their hosts with some notable host jumps, eg. from a rosaceous to an ericaceous host or vice-versa [17]. *Botrytis* (sexual state is *Botryotinia*), includes both host generalists and specialists, and shows a history of host-shifts rather than co-evolution with hosts. There are two main clades of *Botrytis*: one that is able to colonize only eudicots and the other infecting eudicots or monocots [18]. Within the genus *Sclerotinia*, at one extreme *S. sclerotiorum* is arguably the necrotrophic pathogen with the largest host range (virtually any eudicotyledonous plant – with at least 400 species reported), while at the other extreme, *S. glacialis* is associated with one host, *Ranunculus glacialis* [19]. Another important specialized necrotroph is “*Sclerotinia*” *homoeocarpa*, a pathogen of turf (dollar spot) closely related to *Poculum henningsianum*, another grass pathogen, in the Rutstroemiaceae, the sister family to the Sclerotiniaceae. *S. homoeocarpa* is not a true *Sclerotinia*, but has not yet been formally reclassified in another genus in Rutstroemiaceae. Although species in *Botrytis* and *Sclerotinia* are well known necrotrophs, some show periods of latency between infection and development of disease [20], or symptomless endophytism [21], [22].

Our study required an experimentally tractable sample of genes representative of what is undoubtedly a larger “toolbox” of genes contributing to interaction with host plants in symbiosis, inclusive of disease and endophytism/latency. As candidate pathogenicity-related genes, we selected from those that have been investigated specifically as pathogenicity factors in the Sclerotiniaceae. In a more general sense we looked to comparative analyses of completed fungal genomes that have revealed some major differences between biotrophic and necrotrophic fungi [23], [24]. The genomes of the biotrophic pathogens, *Ustilago maydis* and *Blumeria graminis*, and the ectomycorrhizal fungus, *Laccaria bicolor*, have greatly reduced numbers of cell wall degrading enzymes (CWDEs) as compared with genomes of necrotrophs [25]–[27]. Because the presence of CWDEs is hypothesized to characterize necrotrophy, and not biotrophy, we selected some of the CWDE-encoding genes for study. CWDEs not only facilitate penetration, colonization and maceration of the host tissues, but also contribute by generating a nutrient source for the pathogen from components of the host cell wall [28], [29]. We selected genes encoding an aspartyl protease (*asps*) and an acid protease (*acp1*), known to be expressed in the early stages of infection by *S. sclerotiorum*, and to accumulate during lesion expansion and necrosis [30], [31].

The evolutionary arms race model predicts a dynamic between pathogen and host in which fungal pathogenicity factors and plant defense responses are both influenced by positive selection [32], [33]. Consistent with the model, fungal polygalacturonases are specifically recognized and inhibited by polygalacturonase-inhibiting proteins in plants [34], [35]. Polygalacturonases from *S. sclerotiorum* are present early in the infection process and have been implicated in initiating programmed cell death [36], [37]. Mutants of the *B. cinerea* endopolygalacturonase gene, *Bcpg1*, showed reduced colonization of host tissues [38], while a mutant of *Bcpg2*, showed a delay in the development of a primary lesion [39]. We selected *pg1*, *pg3*, *pg5*, and *pg6* for the present study because they have homologues in both *S. sclerotiorum* and *B. cinerea*.

Ubiquitous among filamentous fungi, oxaloacetate acetylhydrolase (OAH and OAH-like enzymes) are a subclass of the phosphenolpyruvate mutase/isocitrate lyase superfamily that catalyse hydrolysis of oxaloacetate to oxalic acid and acetate. Production and secretion of oxalic acid is associated with fungal pathogenesis and virulence [40], [41]. Hypothesized virulence mechanisms include acidification that permits pathogen growth and development, that cues degradation of lignocellulose in wood decay, or that induces crystallization of calcium oxalate which is very common among even non-pathogenic filamentous fungi but among pathogenic fungi can block vessels or bronchioles (eg. *Aspergillus fumigatus*) [42]. Fungal genomes have been shown to encode many OAH homologues [41] with high sequence identity (termed OAH-like), as distinguished from the subset that are active in producing oxalic acid (termed OAH). Among basidiomycetous and ascomycetous fungi encoding OAH-class proteins, conservation of synteny at the OAH locus was demonstrated among *S. sclerotiorum*, *B. cinerea*, *Aspergillus fumigatus*, *A. oryzae* and *A. niger*, although conservation of synteny among other, OAH-like proteins was found only among this group of *Aspergillus* species, exclusive of *S. sclerotiorum* and *B. cinerea*. There is also phylogenetic structure based on analyses of the proteins consistent with patterns of synteny conservation [41]. It is clear that not all OAH-like proteins are the same and that their diversity, as reflected in phylogenetic trees and patterns of synteny conservation, reflects the diversity of the fungi that produce them. An active site, a serine residue at position 281, is common only to oxalate-producing fungal strains, such as characterized strains of *S. sclerotiorum* and *B. cinerea*, and is hypothesized to represent the active catalyst [41].

Oxalic acid, a pathogenicity determinant in *S. sclerotiorum* [43], is also produced at high levels in *B. cinerea* [44]. Effects include enhancement of the environment for expression of CWDEs by lowering pH early in infection [28], [29], suppression of the oxidative burst [45], interference with stomatal closure facilitating ingress [46], regulation of sclerotial formation [47], elicitation of programmed cell death in a manner sharing features with mammalian apoptosis [8], and modulation of host redox status [48]. While we cannot know whether oxalic acid plays all of these roles among related species, or entirely different roles, some functions are likely shared, such providing the acidic environment for sclerotial development (all species in the Sclerotiniaceae form sclerotia) and for activity of secreted enzymes such as pectinases, proteinases, and laccases (reviewed in [49]). Another gene of interest is *pac1*, a zinc finger transcription factor mediating expression of pH-regulated genes [47], [50]. Mutants of *pac1* showed reduced virulence in pathogenicity tests with tomato and *Arabidopsis* and lowered levels of oxalate accumulation [47], [50].

Are differences between trophic types (biotroph vs. necrotroph, host generalist vs. specialist) associated with selection on primary sequence, or with differences in gene expression? We first hypothesized that among species in this strongly supported evolutionary lineage of fairly recent origin, there is a common toolbox of pathogenicity related genes inherited from the most recent common ancestor. The second hypothesis is that given a common toolbox, ability to interact with the host as a necrotroph, biotroph, generalist or specialist depends to a large degree on timing and magnitude of expression of toolbox genes.

Hypotheses were addressed following a chain of inference based on a series of analyses or experiments. 1) To determine whether biotrophy or necrotrophy evolved once or multiple times, trophic choice was mapped on a phylogeny of three, selectively neutral housekeeping genes. 2) To test hypothesis one, the common toolbox, we sought to amplify a common set of pathogenicity-related genes known to be present in *S. sclerotiorum* and *B. cinerea* in a hierarchical sample of genera, species and strains across the family; ability to amplify these genes in all genera sampled would be consistent with a common toolbox. 3) To further investigate hypothesis one by comparing type and strength of selection on the sample set of pathogenicity-related genes, we determined the incongruencies between the housekeeping gene phylogeny and those based on pathogenicity-related genes; incongruencies indicate differing evolutionary histories for species and genes, consistent with selection. Site-specific likelihood analyses, comparing species or trophic type classes, were used to test for purifying selection, indicative of an essential, conserved gene function, and positive selection, consistent with an evolutionary arms race with the host. Branch-specific likelihood analyses tested for differences in rates of selection between trophic types. 4) Hypothesis two, timing and magnitude of gene expression, was tested in three steps. First, to qualitatively compare and infer constitutive presence, absence and timing of a key pathogenicity-associated product, oxalic acid, strains were screened on an indicator medium under controlled conditions. Second, to qualitatively observe ability to infect living plants and timing of infection – on a level playing field – we infected leaves of *A. thaliana*. From this we could infer whether an intuitive assumption that inability to form lesions predicted biotrophy or specialization while ability to form lesions predicted a necrotrophic generalist habit. Last, again on the level playing field of living *A. thaliana* plants, we used quantitative reverse transcriptase PCR (qRT-PCR) to follow expression of two genes associated with the oxalic acid pathway throughout the course of plant infection. Based on the results of this work, there was support for the common toolbox of pathogenicity-related genes sampled, and a striking difference between necrotrophic generalists and all host specialists in the timing and magnitude of expression of a pathogenicity factor essential for symptom development.

## Results

### At least two origins of biotrophy from a necrotrophic ancestor in the Sclerotiniaceae

**Figure 1:**
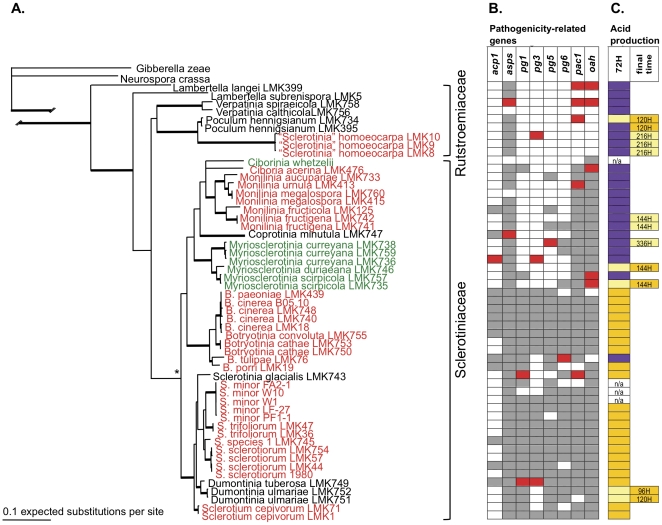
Phylogeny of the Sclerotiniaceae inferred from 3 housekeeping loci showing incongruencies with pathogenicity-related genes trees, and results of acid production on indicator plates. **A.** Housekeeping gene phylogeny of the Sclerotiniaceae from the concatenated sequence of *hsp60*, *g3pdh* and *cal*. The putative obligate biotroph, *Ciborinia whetzelii*, was added based on the placement of Holst-Jensen *et al.* [52]. Thick branches represent well-supported nodes with >90% support from 1000 maximum likelihood (ML) bootstrapped pseudoreplicates and >0.95 posterior probabilities. An asterisk represents the node with >75% ML bootstrap support and >0.90 posterior probabilities. Trophic type is designated by text color: necrotrophs in red, biotrophs in green, and taxa with unknown life histories in black. **B.** Incongruence between the housekeeping gene phylogeny in Figure 1A and the phylogeny of each pathogenicity-related gene (*acp1*, *asps*, *pg1*, *pg3*, *pg5*, *pg6*, *pac1*, *oah*). Horizontally aligned with the housekeeping phylogeny, red fill designates a species incongruent with respect to the gene indicated at the top of the column and the housekeeping phylogeny; grey fill indicates congruence; white fill represents a gene that failed to amplify with the primers employed. **C.** Results from bromophenol blue plates indicating acid production as a proxy for oxalic acid production at two time points: 72 hours post-plating, and a final time point if acid production was delayed. Purple fill represents no color change indicating no acid production or production below the limit for detection, bright yellow fill represents a strong color change qualitatively indicating higher production, and a light yellow fill represents a less intense color change. Isolates not tested are n/a.

The Bayesian phylogeny inferred from the combined sequence of the housekeeping loci (*cal*, *hsp60* and *g3pdh*) is shown with nodes supported by joint ML bootstrap and posterior probabilities (Figure 1A). The total aligned sequence length for the three housekeeping genes was 2181 characters, of which 922 were parsimony-informative. The representative phylogeny inferred from housekeeping genes revealed a clear genetic distinction between the Rutstroemiaceae and Sclerotiniaceae, as well as well-supported clades for all major genera represented (Figure 1A). The topology is consistent with previously published works at the rank of family [51], and genera and species [52]. The obligate biotroph, *C. whetzelii*, was added to the phylogeny based on the placement of Holst-Jensen *et al.* [52]. When the character of trophic type is mapped onto the phylogeny, the most parsimonious interpretation is that the common ancestor of the Sclerotiniaceae was necrotrophic, with biotrophy the derived state, given at least two shifts from necrotrophy to biotrophy.

### Conflict between pathogenicity-related gene trees and the phylogeny inferred from housekeeping genes

Figures 1A and 1B show the incongruencies in tree topology between the housekeeping gene tree and phylogenies for each of the 8 pathogenicity-related genes (*pac1*, *oah*, *acp1*, *asps*, *pg1*, *pg3*, *pg5*, and *pg6*). Incongruencies suggest that the evolutionary histories of all examined genes are discordant with the presumed evolutionary history of the organisms. Given that the arms race model predicts that pathogenicity-related genes evolve under selection imposed by active interaction with host, incongruence in tree comparisons could be due to selection. Alternatively, the incongruence could be the result of recombination, lineage sorting or gene duplication [53]. We explicitly tested for evidence of positive or purifying selection on the pathogenicity-related genes using likelihood-based models of codon substitution with results reported in the following section.

### Site-specific likelihood analyses show evidence for purifying selection acting on all pathogenicity-related genes, and positive selection on sites within 5 of 8 genes

The distribution of the ratio of non-synonymous to synonymous changes across all sites was estimated for the codon alignment of 8 pathogenicity-related genes and 2 housekeeping genes (Table 1). The site-specific likelihood analyses, using the M8-M7 comparison (see Materials and Methods), provided evidence for positive selection acting on sites within 5 of the 8 pathogenicity-related genes (Table 1), consistent with the prediction of the evolutionary arms race model. The housekeeping genes are highly conserved and show no evidence of positive selection at any sites. The data for all models, including parameter estimates and positively selected sites, are shown in Table S1. There was significant variation in the rates among sites for all genes, and most sites were under strong purifying selection for all models examined, indicated by ω values (*d*_N_/*d*_S_) that were generally much lower than 1 (Table S1). The *oah* gene, as well as *pg1* and *pg6*, were highly conserved across all sampled taxa in which they could be amplified and were under strong purifying selection (Table S1), with no sites experiencing positive selection (Table 1).

**Table 1:** Site-specific likelihood analyses for eight pathogenicity-related genes and two housekeeping genes.

Gene	Model	lnL	*P*-value from LRT	Significance
*acp1*	M7	−2278.428308	9.700E^−06^ *	Evidence for positive selection with approx. 0.4% of sites
	M8	−2266.884978		
*asps*	M7	−8489.185656	5.780E^−05^ *	Evidence for positive selection with approx. 3% of sites
	M8	−8479.427215		
*oah*	M7	−2278.428308	0.997	No evidence for positive selection
	M8	−2266.884978		
*pac1*	M7	−6061.749994	0.032 *	Evidence for positive selection with approx. 0.2% of sites
	M8	−6058.318444		
*pg1*	M7	−2547.035662	0.898	No evidence for positive selection
	M8	−2546.928491		
*pg3*	M7	−3954.840538	0.041 *	Evidence for positive selection with approx. 1.8% of sites
	M8	−3951.653571		
*pg5*	M7	−3901.506352	5.894E^−4^ *	Evidence for positive selection with approx. 4.3% of sites
	M8	−3894.069947		
*pg6*	M7	−3978.435472	0.217	No evidence for positive selection
	M8	−3976.906914		
*g3pdh*	M7	−3339.145488	0.999	No evidence for positive selection
	M8	−3339.145248		
*hsp60*	M7	−5604.523767	0.999	No evidence for positive selection
	M8	−5604.524054		

A likelihood ratio test (LRT) was performed to compare the likelihood scores (lnL) of the two nested models: a model that accounts for sites with ω>1 (M8) and a null model that does not (M7). *P*-values that are statistically significant are indicated with an asterisk.

### Branch-specific likelihood analyses show no differences in rates of selection between necrotrophs and biotrophs, and host generalists and host specialists

The likelihood values and the LRT results of the branch-specific likelihood analyses are shown in Table 2, while the parameter estimates and positively selected sites for all models are given in Table S2. There was little evidence for substitution rate differences between trophic types, as only *pg6* showed any difference between biotrophs and necrotrophs (Table 2A). There was no evidence for substitution rate differences between host-generalists and host-specialists (Table 2B).

**Table 2:** Branch-specific likelihood analyses for lineage comparisons.

		A.		B.	
Gene	Model	lnL	*P*-value from LRT	lnL	*P*-value from LRT
*acp1*	Null	−2282.941522	1	−2279.105981	1
	Alternative	−2282.941522		−2279.105981	
*asps*	Null	−8532.680653	1	−8529.262603	1
	Alternative	−8532.680653		−8529.262603	
*oah*	Null	−4187.390913	1	−4174.559675	1
	Alternative	−4187.390913		−4174.559675	
*pac1*	Null	−6083.830891	1	−6067.333617	1
	Alternative	−6083.830891		−6067.333617	
*pg1*	Null	−2542.400238	0.958	−2542.400236	0.958
	Alternative	−2542.398874		−2542.398873	
*pg3*	Null			−3954.533847	0.998
	Alternative			−3954.533843	
*pg5*	Null			−3901.085817	1
	Alternative			−3901.085817	
*pg6*	Null	−3980.295815	0.022 *	−3977.686693	1
	Alternative	−3977.667451		−3977.686693	

The null model fixes the *d*N/*d*S ratio across all lineages in the phylogeny, while the alternative model allows for a different ω value for the foreground branch(es). *P*-values that are statistically significant are indicated with an asterisk.

A Comparisons of the biotrophic lineage(s) to all other necrotrophic lineages.

B Comparisons of all host-specialist lineages to host-generalist lineages in the Sclerotiniaceae.

### Necrotrophic generalists caused early lesion formation and expansion on detached *A. thaliana* leaves, while host specialists and biotrophs either failed to produce disease lesions, or formed small lesions later in the infection process

**Figure 2:**
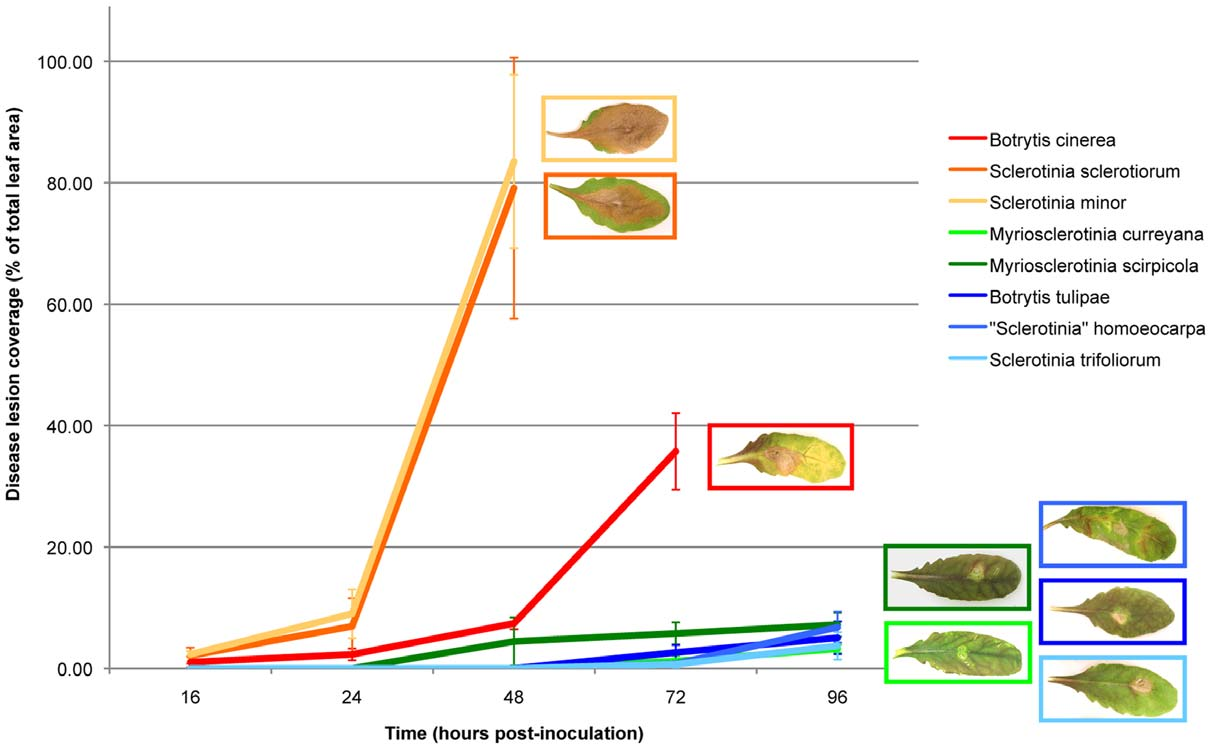
Relative virulence of fungal strains on detached *Arabidopsis thaliana* leaves. Disease lesions were measured as a percentage of total leaf area at 16, 24, 48, 72 and 96 hours post-inoculation using ImageJ. Error bars on both graphs represent the standard deviation among 3 biological replicates. Necrotrophic host generalists are shown in warm colors (reds and oranges), while necrotrophic and biotrophic host specialists are shown in cool colors (blues and greens, respectively). Strains that did not produce disease lesions on *A. thaliana* (*Monilinia aucupariae*, *Monilinia fructicola*, *S. glacialis*, and *Sclerotium cepivorum*) are not shown.

The percentage of leaf covered by disease lesions was used as the metric for relative virulence of strains screened (Figure 2). The necrotrophic generalists, *S. sclerotiorum*, *S. minor* and *B. cinerea*, caused large proliferating lesions within the first 72 hours post-inoculation. Species classified as necrotrophic or biotrophic host specialists, *S. homoeocarpa*, *S. trifoliorum*, *B. tulipae*, *Myriosclerotinia curreyana*, and *M. scirpicola*, caused much smaller disease lesions that were not evident until 96 hours post-inoculation. Host specialists, *Monilinia aucupariae*, *M. fructicola*, *S. glacialis*, and *Sclerotium cepivorum* did not produce lesions on *A. thaliana*.

### Screened for constitutive acid production on indicator plates, most strains produced oxalic acid, but differed in the timing

All strains tested of *Sclerotinia*, *Dumontinia*, *Sclerotium cepivorum* and *Botrytis* produced the characteristic strong yellow color change, with the one exception of *B. tulipae* (Figure 1C). The color change was inconsistently observed among strains of *Monilinia and Myriosclerotinia*. In *Monilinia*, it was observed only in the two strains of *M. fructigena* but not in other species including *M. fructicola*. In *Myriosclerotinia*, it was observed by 72 hours in one of two strains *M. scirpicola* and one strain of *M. duriaeana*, and very late in one of three strains of *M. curreyana* (Figure 1C). *Coprotinia minutula*, which produces apothecia on herbivore dung and may be a biotrophic, symptomless, endophyte, effected no color change. All of the sampled Rutstroemiaceae are thought to be host specialists (as are the majority of species in the family) based on associations of symptoms or sexual fruiting bodies with host plants. The aggressive turf pathogen, *S. homoeocarpa* uniformly effected color change late (216 hours), and its close relative, *Poculum henningsianum*, early (72–120 hours). The dicot-infecting Rutstroemiaceae did not effect color change. It is possible that acid production in some strains was below the detection limit of the color change from purple to yellow.

### *oah* and *pac1* express constitutively but modulation in magnitude and timing is induced

**Figure 3:**
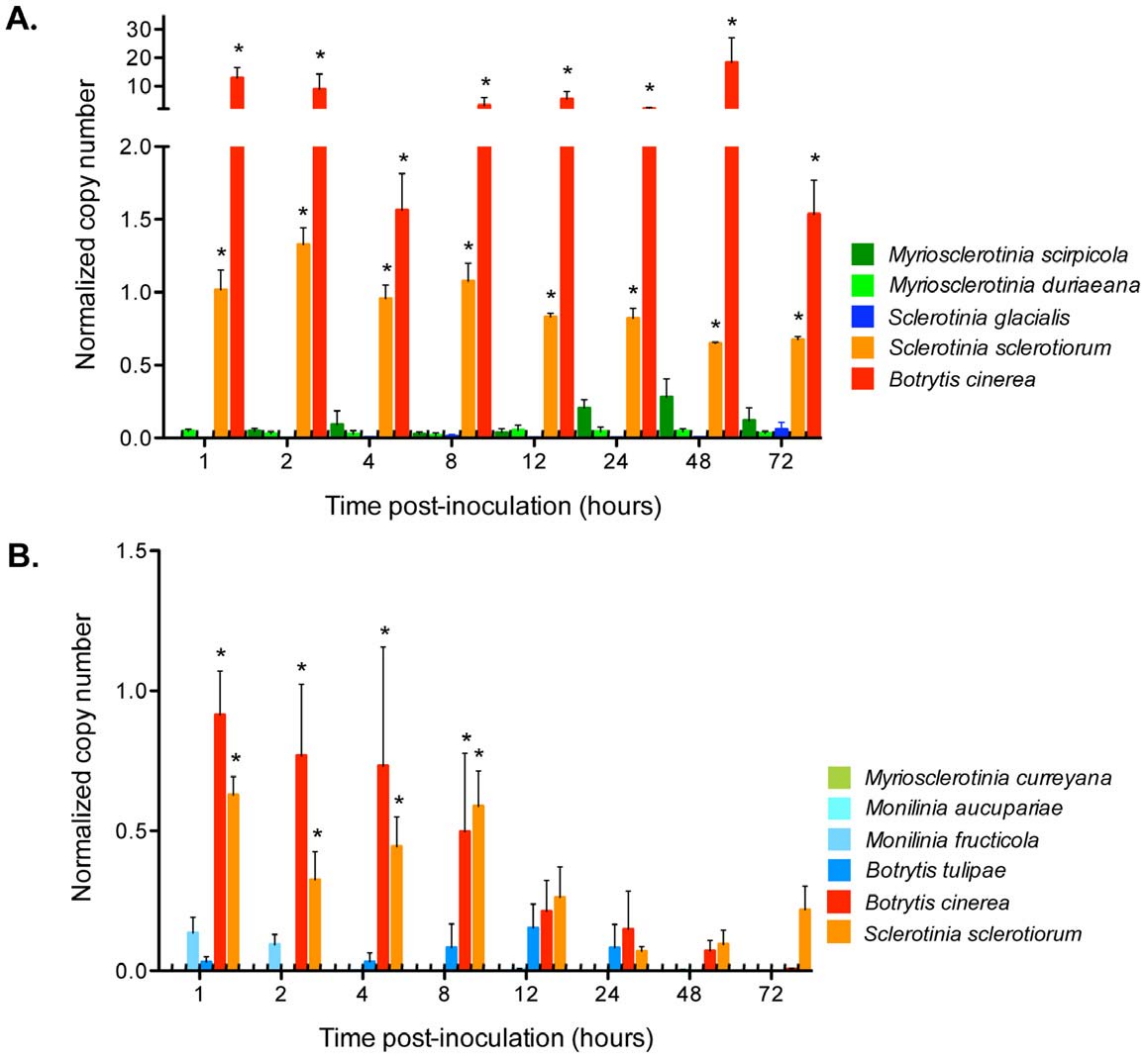
*In planta* normalized *oah* transcript copy number at sampling times 1, 2, 4, 8, 12, 24, 48 and 72 hours post-inoculation. All *oah* gene expression values are represented normalized to the housekeeping gene, *actin*. Error bars on both graphs represent the standard error among 3 biological replicates. Asterisks represent values that are significantly different using a split plot factorial repeated measures analysis. **A.** The first trial included the necrotrophic generalists, *Botrytis cinerea* and *Sclerotinia sclerotiorum*, a host specialist of unknown trophic type, *S. glacialis*, and the biotrophic specialists, *Myriosclerotinia scirpicola* and *Myriosclerotinia duriaeana*. **B.** The second trial included the necrotrophic generalists, *Botrytis cinerea* and *Sclerotinia sclerotiorum*, the necrotrophic specialists, *B. tulipae*, *Monilinia fructicola*, and *Monilinia aucupariae*, and the biotrophic specialist, *Myriosclerotinia curreyana*.

Within the first 4 hours of plant inoculation, *in planta oah* expression levels, normalized to *act*, were 10 to 300 times greater among necrotrophic host generalists than among biotrophs and host specialists; *pac1* levels did not vary in association with trophic type or host generalist/specialist habit (Figure 3). In the first trial (Figure 3A), *B. cinerea* (strain B05.10) showed significant differences in the magnitude of expression of the *oah* gene compared to all others (*P* = 0.000). The magnitude of gene expression was so great for *B. cinerea* that it obscured statistical differences among all other strains. When *B. cinerea* data were removed from the dataset, *S. sclerotiorum* showed significant differences in the magnitude of *oah* expression compared to the other remaining taxa (*P* = 0.039). In the second trial, the *oah* expression of *B. cinerea* and *S. sclerotiorum* was significantly different (*P* = 0.009) from all other taxa at early time points, 1 to 8 hours post-inoculation (Figure 3B). There was a 10- to 30-fold difference in the level of *oah* expression for *B. cinerea* strain B05.10 between the two trials. These trials are independent experiments and although both were conducted under controlled conditions, maturity of plant tissue and plant physiology cannot be completely standardized. The early time points were close to one another, and it is possible to have hit or missed expression peaks during the sampled times. Comparing the two trials, trends were consistent and statistically robust. Expression of *oah* and *pac1* was detected in RNAs from mycelium grown *in vitro*, indicating that *oah* and *pac1* are constitutively expressed. There was no expression of *oah*, *pac1* or *act* in any mock-inoculated control.

## Discussion

Our data indicate that among a sample of pathogenicity-related genes, there is a common toolbox of genes under similar types and strengths of selection shared by members of the Sclerotiniaceae. This hypothesis is supported by the amplification of 6/8 pathogenicity-related genes in all genera, although not in all species in a genus. Interestingly, this includes genes encoding cell wall degrading enzymes (*acp1*, *asps*, *pg1*, *pg3*, *pg5* and *pg6*) in biotrophic *Myriosclerotinia* species, supporting phylogenetic evidence (Figures 1A, 1B) that biotrophy in the Sclerotiniaceae is derived from necrotrophy and may represent a type of necrotrophy. Given our expectation that selection on pathogenicity-related genes would result in incongruence with the phylogeny inferred from neutrally evolving housekeeping genes, incongruence was surprisingly limited. Although there was evidence for positive selection on sites within 5 of the 8 pathogenicity-related genes, there was little to no evidence that the rates of selection differed among trophic types.

The primary sequence of all pathogenicity-related genes studied is fairly well conserved due to strong purifying selection. The *oah* gene is a striking example of this, with a high level of conservation across all sampled taxa, including the obligate biotroph, *C. whetzelii*. That the *oah* gene is under strong purifying selection, with no sites under positive selection, is evidence that the gene product plays an important role in interactions with the host plant resulting in nutrient acquisition among members of the Sclerotiniaceae. This is a key piece of evidence for the common toolbox hypothesis. Although the role of oxalic acid has been well-investigated in *S. sclerotiorum* and *B. cinerea*, the results presented here indicate that the potential to produce oxalic acid, essential to full symptom development in *S. sclerotiorum* and therefore important in virulence, is shared among many host-specialized or poorly known species in the Sclerotiniaceae – and was likely inherited from their common ancestor.

Based on the phylogeny inferred from loci assumed to be neutral, the common ancestor of the Sclerotiniaceae was necrotrophic, and there were at least two shifts from necrotrophy to biotrophy. Surprisingly, there were very few differences between this phylogeny and those inferred from 8 pathogenicity-related genes (Figure 1B, Figures S1, S2, S3, S4, S5, S6, S7, S8). To explain this limited incongruence, we looked for evidence of positive selection on the pathogenicity-related genes, as predicted by an evolutionary arms race model. Five of the 8 pathogenicity-related genes had sites with the signatures of positive selection. But all pathogenicity-related gene trees showed some incongruence, so factors other than selection should be considered, such as recombination, lineage sorting or gene duplication [53]. Lateral gene transfer is unlikely, as all pathogenicity-related gene sequences are similar to one another, indicative of common ancestry.

The evidence for positive selection suggests that these genes are involved in a co-evolutionary interaction with the host defense response. While we detected no positive selection in *pg1*, previous studies among strains of *B. cinerea* provided evidence consistent with positive selection on both *pg1* and *pg2* [54], [55]. Direct comparisons to the previous work were not possible because of critical missing information, specifically the model of codon evolution [54], and the *d*_N_/*d*_S_ values [55]. These intraspecific, population scale studies in *B. cinerea* address a different question than ours. They capture dynamic evolutionary processes as new alleles are emerging and competing before becoming fixed. We were looking at a different evolutionary scale, in which alleles conveying a selective advantage among lineages with different trophic preferences would likely have become fixed.

Results from our work and previous studies suggest that the role that polygalacturonases play during pathogenesis may be different among members of the Sclerotiniaceae. For example, the *pg2* gene is important in pathogenesis for *B. cinerea* [39], but has no homologue in *S. sclerotiorum*. We were able to amplify all of the polygalacturonase-encoding genes from *Myriosclerotinia* (but not from all species or strains) and amplified only two from *Monilinia* strains. We cannot determine whether this means that the genes are absent, as with *pg2* in *S. sclerotiorum*, or that the sequences vary at primer sites. Such variability is suggested by the result that all polygalacturonase-encoding genes can be amplified among species of *Myriosclerotinia*, but the same suite of genes cannot be amplified from all species or from all strains representing a species.

Despite the evidence for positive selection acting on some of the pathogenicity-related genes, there was little to no difference in substitution rates between biotrophs and necrotrophs, and host specialists and generalists. That substitution rates in pathogenicity-related loci were not associated with trophic type or host specialization in our study is consistent with the finding of Rowe and Kliebenstein [54] that sequence variation at *pg1* and *pg2* was not associated with virulence on *A. thaliana*.

In the present study, from all species in the Sclerotiniaceae studied, including necrotrophic generalists and specialists, and biotrophic specialists, all amplicons of the *oah* gene included the proposed active marker, the serine residue at position 281 [41]. None amplified an inactive homologue. From this, it is likely that all sampled species, a wide sample of the Sclerotiniaceae and two species from the Rutstroemiaceae, are producing oxalic acid, especially with the additional evidence from the screen on bromophenol blue indicator plates. The most likely scenario is that oxalic acid is fulfilling expected functions among this lineage of closely related species, but the full range of functions cannot be known here.

Distinction among trophic types was seen in the timing and magnitude of oxalic acid expression both *in vitro* and during plant infection. Indicator plates showed that most strains used in our screen produced oxalic acid, but differed in the timing of production *in vitro*. All necrotrophic generalists produced oxalic acid by 72 hours post-inoculation, while production was usually either absent or delayed among necrotrophic and biotrophic specialists. This pattern was more precisely observed and pronounced in the *in planta* gene expression experiment in which abundant expression of *oah* was observed within 8 hours of inoculation only among necrotrophic generalists.

There is general agreement between both assays for oxalic acid production, with the exceptions of *M. fructicola* and *B. tulipae*. Both strains showed no color change on indicator plates, while *oah* transcripts were expressed at low levels. There was constitutive expression of *oah* in pure fungal RNAs. Given this, it is more likely that oxalic acid production was below the detection limit of the indicator plate assay than that it was absent in plates and induced only during the host interaction. That the timing and magnitude of *oah* was differentially expressed between necrotrophic generalists and necrotrophic or biotrophic host specialists was consistent with our predictions. Our results agree with the evidence provided by Wang *et al.* [56], Dickman and Mitra [43] and Williams *et al.* [48] that oxalic acid is an important pathogenicity factor in *S. sclerotiorum*.

Alternative to our contention that given that oxalic acid is produced across the closely related species of the Sclerotiniaceae, it is in the common toolbox for trophic interaction and nutrient acquisition, it could be argued that early, high *oah* expression in pathogenesis is specific to *S. sclerotiorum* and *B. cinerea* – that other species interact with their host plants via other, as yet unknown mechanisms and that oxalic acid has other roles. This is possible. The objective of the present study, however, was to screen expression of a likely pathogenicity factor as a kind of taxonomic character. On the level playing field of a controlled experiment on one host, *A. thaliana*, over a defined time scale, was there a predictive, repeatable response? Yes, there was, with necrotrophic generalists expressing early and high, and specialists, late and low (one species did not express). Such a screen could be run for each species on its real host, but it would not be standardized and would be logistically difficult. We see this study as a baseline for future, more species-specific comparisons. The present study provides direction for selecting species to be compared in such studies.

All generalist necrotrophic strains initiated the early formation of primary lesions, followed quickly by secondary lesion expansion. All specialist and biotrophic strains either failed to cause disease lesions, or produced smaller lesions at later time points. None of the specialists are known to infect the Brassicaceae. The ability to infect and colonize detached leaves of *A. thaliana*, coupled with the ability to produce oxalic acid *in vitro* by 72 hours and *in planta* by 8 hours, distinguished all necrotrophic generalists that we screened.

The wild plant-infecting, host specialists of the Sclerotiniaceae are rarely collected and are poorly represented in institutional culture collections. The phenology of fungus and plant is key to finding fruiting bodies and to witnessing development of the plant interaction; few master this knowledge and are fortunate enough to find these often-rare species. Collectors see obvious spore-producing structures and deduce plant associations. For example, in many species in the Sclerotiniaceae, fruiting bodies are produced on or around putative host plants, but since ascospores or conidia are the primary inoculum, disease development occurs at a later time and endophytic or latent phases are virtually invisible unless plant tissue is cultured. Indeed we hope that current large-scale projects to isolate and identify plant endophytes world-wide [57] will turn up more isolates of the Sclerotiniaceae and reveal more about host range and trophic type of these and other fungi. One goal of the present research was to use our culture collection to find alternative approaches that shed light on these life histories. Within the Sclerotiniaceae, we have distinguished necrotrophic generalists from biotrophs and host specialists by the ability to produce abundant oxalic acid and to cause large proliferating lesions on detached leaves of *A. thaliana*. We can apply these criteria for categorization of taxa with unknown life histories. For example, *S. glacialis* has only been found in association with *Ranunculus glacialis* [19]. Further evidence of host specialization in our study was the late and low *oah* expression *in planta*, and the failure to produce disease lesions on *A. thaliana*. That *S. homoeocarpa* has the signature of a facultative biotroph or specialist with late expression of oxalic acid (Figure 1C) is important in better conceptualizing its role as a pathogen and devising better management of dollar spot in turf. It is also further evidence of its distinction from *Sclerotinia* and the Sclerotiniaceae. The similar pattern in *Poculum henningsianum* with its relatively unexplored life history but close relatedness to *S. homoeocarpa* was an especially enlightening result. There are differences in oxalic acid production among the biotrophic *Myriosclerotinia* species. *M. scirpicola* had late and low *oah* expression, and a delay in oxalic acid accumulation *in vitro*. In contrast, *M. curreyana* had no *oah* expression and did not produce detectable levels of oxalic acid. Interestingly, both species could infect and cause small lesions on detached leaves of *A. thaliana*. We hypothesize that *Myriosclerotinia* species, though evolved from a necrotrophic ancestor, may be constrained in their host rushes, sedges and grasses by mechanical host defenses – sclerenchyma, lignified bundle sheaths or silica bodies in the epidermis [15], [58]. Evidence for biotrophy in *M. curreyana* includes the retention of chlorophyll in sites of sclerotial formation, low in vitro cellulytic enzyme production, and suppression of pectinolytic enzymes by 1% glucose [59].

The results of the present study identify candidate species for whole-genome sequencing. The question remains whether genomes of presumptive biotrophs in the Sclerotiniaceae, *Myriosclerotinia* species, or *Coprotinia minutula*, or *Ciborinia whetzelii* share the expansions of plant cell wall degrading enzyme gene families with necrotrophs in the Sclerotiniaceae, such as *B. cinerea* and *S. sclerotiorum*. Alternatively, but less plausibly, these presumptive biotrophs in the Sclerotiniaceae may follow the precedents of the exemplars, the basidiomycotans, *Ustilago maydis* and *Laccaria bicolor*, and fellow Leotiomycete, *Blumeria graminis*, with the reduction of proteins involved in plant cell-wall degradation and secondary metabolites, and the expansion of secreted effector proteins hypothesized to evade or suppress the plant defense response [25]–[27]. Based on our results, the expectation is that the presumptive biotrophs in the Sclerotiniaceae share a form of endophytism derived from necrotrophic ancestors in the family – that they are constrained by the host or experience periods of endophytism with the potential for causing disease.

We must understand alternative paths that deviate from the exemplars of biotrophy or necrotrophy in order to understand the evolution of trophic choice among different fungal lineages, likely with different toolboxes for host interaction. The genomic criteria for categorizing trophic types might not pertain at the scale of the family, where differences between biotrophs and necrotrophs, and generalists and specialists, might represent a continuum of trophic interactions. We would expect the genomes of host specialist species in the Sclerotiniaceae to be similar to the genomes of *S. sclerotiorum* and *B. cinerea* due to shared common ancestry, and the evidence for a common toolbox. We might expect lineage-specific differences that account for host specialization. For example detoxifying plant defense compounds, such as phytoalexins, as an infection strategy for host generalists where defense compounds are general in effect, or for specialists when the defense compounds are specialized in effect [10], [11]. Additionally, and especially pertinent to data presented here, we might also expect differences at the scale of transcriptional regulators of gene expression.

Host infection is a complex process requiring a network of interactions, some incorporating functional redundancy, among pathogenicity-related genes as well as global regulators and epigenetic factors. Knockout and gene complementation experiments operate in this complex landscape. To date, most of the work assessing the role of putative pathogenicity genes has been accomplished using gene knockouts. Genes are characterized as virulence factors when inactivation of the gene renders the pathogen less virulent, and complementation restores original gene function. Based on knockouts followed by complementation, the evidence suggests that genes involved in signaling are the key, in fact the only, demonstrable pathogenicity determinants in *B. cinerea*, i.e. *bmp1*, encoding a map kinase protein gene ([60], reviewed in [61]). In *S. sclerotiorum*, targeted gene deletion mutagenesis of the *oah1* gene, complemented by re-introduction of the wild-type copy of *oah1* has successfully demonstrated 1) the key role of oxaloacetate acetylhydrolase encoded by *oah1* in oxalic acid biosynthesis and 2) that oxalic acid is required for full symptom development but not absolutely required for pathogenesis (J. Rollins, pers. comm.). In the present study, comparative *in vitro* and *in planta* expression sheds further light on the genetic mechanisms underlying trophic types across an evolutionary lineage of species, the Sclerotiniaceae. The need for this course of investigation was pointed out in “Genomic analysis of the necrotrophic pathogens, *Sclerotinia sclerotiorum* and *Botrytis cinerea*” [62], the comparative analysis of annotated genomes for these exemplars of necrotrophy. Our results suggest that timing and magnitude of *oah* gene expression underlies differences between host generalists and specialists, but not biotrophs and necrotrophs. Alternatively, there may be factors unique to the host specialists or biotrophs as yet unknown; finding and understanding such specific interactions will require whole genomes and transcriptomics within both the actual host and a model host with better supporting resources. This is a challenging prospect for a comparative study of a lineage of species, although perhaps a way of getting at the signature of necrotrophy still somewhat elusive even in the wake of the *Sclerotinia/Botrytis* genome project [62].

Comparative expression studies with additional potential or known pathogenicity factors would be needed to determine how generally important differences in timing and magnitude are to trophic outcomes in the host within this or other fungal family lineages. Candidates include the pathogenicity-related genes that encode CWDES and are pH-regulated, such as the hydrolytic polygalacturonases. Would these follow similar trajectories to that observed for *oah* expression, given that the regulation of oxalic acid plays an important role in modulating pH throughout the course of plant infection? Last, how is expression regulated? It is likely by networks not unique to trophic types or to the Sclerotiniaceae.

Can we better distinguish or understand the fundamental nature of necrotrophs and biotrophs in the Sclerotiniaceae? The defense response of the host counters expression of the pathogenicity toolbox. Given this, monitoring the expression of key genes associated with JA and ethylene signaling (*DDE2* and *EIN2*), and SA signaling (*SID2*) could use the plant to discriminate between necrotrophic or biotrophic infection – or detect the kind of necrotrophy in biotrophy's clothing that we may be seeing in the Sclerotiniaceae, if for example, a symptomless endophyte and presumed biotroph such as *Myriosclerotinia* incites JA signaling in the plant [63]. In another approach, quantifying host and pathogen transcriptomes through the course of plant infection should elucidate what mechanisms (in either the host or pathogen) underlie the differences among necrotrophs and biotrophs in this or other fungal lineages.

## Materials and Methods

### Strains used in this study

The test sample included a panel of 52 strains representing 30 taxa (Table S3) of host generalists and host specialists, and the spectrum of trophic types in the families Sclerotiniaceae and Rutstroemiaceae. The sample is anchored by two of the three strains from the *Botrytis/Sclerotinia* genome sequencing project, *B. cinerea* strain B05.10 and *S. sclerotiorum* strain 1980 (http://www.broadinstitute.org/annotation/genome/botrytis_cinerea/Home.html, http://www.broadinstitute.org/annotation/genome/sclerotinia_sclerotiorum/MultiHome.html).

### Fungal strains and DNA extraction

All strains were grown on potato dextrose agar (PDA; Difco Laboratories, Detroit, MI) for 3–5 days, and were then transferred to standing cultures in potato dextrose broth (Difco Laboratories) for 1–2 days. On both solid and liquid media, strains were grown in the dark at ambient room temperature (20–22°C). Cultures were filtered through Miracloth (Calbiochem, EMD Chemicals Inc., Darmstadt, Germany) and lyophilized. DNA was extracted from 10–15 mg of lyophilized mycelium using a DNeasy Plant Mini Kit (Qiagen, Mississauga, ON). Quantity of DNA was estimated on 1.0% ethidium bromide-stained agarose gels, with known quantities of bacteriophage lambda DNA as standards against which comparisons of band intensity could be made. DNA extractions were diluted to 10–20 ng/µL in elution buffer (10 mM Tris-Cl, 0.5 mM EDTA; pH 9.0) for use in PCR.

### PCR and sequencing

The housekeeping genes that were amplified and sequenced were glyceraldehyde-3-phosphate dehydrogenase (*g3pdh*), heat shock protein 60 (*hsp60*), and a 500 bp segment of the calmodulin gene (*cal*). The pathogenicity-related genes code for proteins associated with cell wall degradation: acid protease 1 (*acp1*), aspartyl protease (*asps*), and polygalacturonases (*pg1, pg3, pg5, pg6*), and the oxalic acid pathway: oxaloacetate acetylhydrolase (*oah*), and zinc finger transcription factor (*pac1*). Primer3 [64] was used to design primers for all loci with optimized annealing temperatures ranging from 50°C to 60°C (Table S4). Degenerate primers were designed to amplify across the entire family using the sequence data from the genome strains of *B. cinerea* and *S. sclerotiorum*. All genes were confirmed to be single copy in the genome strains. Amplicon sizes are indicted in Table S5.

Polymerase chain reactions were performed using GoTaq Colourless Master mix (Promega Corporation, Madison, WI) containing: GoTaq DNA Polymerase in 1X Colorless GoTaq Reaction Buffer (pH 8.5), 200 µM dNTPs, and 1.5 mM MgCl_2_; 0.2 µM of each primer; 10–20 ng of DNA template in the total volume of 50 µL per reaction. PCR was performed under the following conditions in a GeneAmp PCR System 9700 programmable thermal cycler (Applied Biosystems, Foster City, CA): denaturing at 95°C for 2 min followed by 35 cycles of 30 s denaturing at 95°C, 30 s annealing at corresponding temperatures for each primer set (Table S4), and 1 min elongation at 72°C, followed by a final extension step at 72°C for 5 min. PCR fragments were visualized by electrophoresis on a 1.0% ethidium-bromide stained agarose gel, using a 100 bp ladder (BioShop Canada Inc., Burlington, ON) to estimate the size of the fragments. All PCR products were purified and sequenced using 3730xl DNA Analyzer systems (Applied Biosystems, Foster City, CA) at the McGill University and Genome Quebec Innovation Centre. Sequences have been deposited in GenBank under accession numbers JQ035790 to JQ036168.

### DNA sequence alignment and phylogenetic analyses

Sequences were aligned in ClustalX v. 1.81 [65] and trimmed manually to the sequence lengths in Table S5. Maximum parsimony (MP) and maximum likelihood (ML) analyses were performed in PAUP* 4.0b10 [66] using heuristic searches for each of the housekeeping and pathogenicity-related loci. Bootstrap support was estimated for internal branches from 1000 pseudoreplicates for MP and ML analyses. Models of sequence evolution were estimated using ModelTest 3.7 [67], [68], and are listed for all loci in Table S5. Bayesian analyses were performed in MrBayes v.3.0b4 [69], to estimate the posterior probabilities of tree topology using Metropolis-coupled Markov chain Monte Carlo (MCMCMC) methods. Independent analyses of each locus were conducted with 3 million generations each, with a sampling frequency of 1 tree every 100 generations. The average standard deviation of split frequencies stabilized (to a difference of less than one percent) after 10000 generations in all our analyses. Therefore the initial 10000 generations from each run were discarded as burn-in when summarizing tree parameters and topology. Priors for the substitution rates were set to a flat Dirichelet distribution.

Tree topologies of the housekeeping genes (*g3pdh*, *hsp60*, and *cal*) were compared for congruence using the Shimodaira-Hasegawa test [70], as implemented in PAUP* 4.0b10 [66]. To ensure that only well-supported nodes were compared, the individual tree topologies were assessed using a cut-off of 70% bootstrap support at all nodes. The SH tests revealed no significant incongruence between the tree topologies of the three loci, and so the individual datasets were concatenated. Analysis of the combined dataset was performed using a partitioned dataset with each partition corresponding to each genetic locus with a unique evolutionary model for each partition using MrBayes v.3.0b4 [69]. Well-supported nodes were defined by >90% support from 1000 ML bootstrapped pseudoreplicates and >0.95 Bayesian posterior probabilities. Supported nodes were defined by >75% ML bootstrap support and >0.90 Bayesian posterior probabilities. Homologous sequences for all housekeeping loci were retrieved for *Gibberella zeae* and *Neurospora crassa* from GenBank [71], and were used to root the phylogeny. Each pathogenicity-related gene tree was compared to the inferred housekeeping gene tree for incongruence, which we defined to be the placement of any taxon in a well-supported clade that differed from the phylogenetic placement in the housekeeping tree.

### General method of screening for positive selection using PAML

A sequence of analyses were used to test for positive selection acting on pathogenicity-related genes using the PAML software package [72], [73]. First, a ML phylogeny was inferred from sequence data for each of the 8 pathogenicity-related genes, and 2 housekeeping genes, in which we assume that allele frequencies between lineages are fixed and not in flux. Calmodulin data were not included for these analyses, as only a partial fragment of the entire gene was amplified. Then, nucleotide sequences were translated into codons using MEGA version 4.0 [74], based on comparative alignment of the genome strains of *S. sclerotiorum* and *B. cinerea*. The distribution of the ω-ratio (*d*_N_/*d*_S_) across sites was estimated for the codon alignment of each gene using different models of codon substitution. Finally, likelihood ratio tests (LRTs) were used to compare the likelihood scores between nested models (models having the same parameters except one model accounts for sites under positive selection and the null model does not).

### Detection of positive selection: Site-specific selection analyses

Site-specific likelihood analyses were used to test for positive selection on specific sites within the pathogenicity-related genes, as predicted by the evolutionary arms race model. Six different models of codon substitution were used: M0, M1a, M2a, M3, M7 and M8 [73], [75]. The LRT between M0 and M3 tested for rate heterogeneity among sites, while the LRT between M1a and M2a, and between M7 and M8, tested for evidence of positive selection. We used the M8-M7 comparison to assess positive selection because it is a more stringent test when a large fraction of sites have 0<ω<1, as was evident in our datasets [76]. The cutoff chosen for significance was *P* = 0.1, which is acceptable because this likelihood ratio test is very conservative [76].

### Detection of positive selection: Branch-specific selection analyses

Branch-specific likelihood analyses were used to determine if the ω-ratio was different in 2 iterations [77]. In one, the foreground branches were biotrophic lineages, and the background branches were necrotrophic. In the second, the foreground branches were host specialist lineages, and the background branches were host generalists. LRTs were used to compare the null model, which fixed the ω-ratio across the phylogeny, with an alternate model that allowed for a free estimation of the ω-ratio along specified foreground branches of the phylogeny. Genes *pg3* and *pg5* were eliminated from iteration one because they could not be amplified in *Myriosclerotinia*. Branch-sites models were also used to detect positive selection that affects a few sites along each lineage, using a Bonferroni correction to account for testing multiple branches [78], [79].

### Virulence testing

Taxa from all major branches in the phylogeny of the Sclerotiniaceae and Rutstroemiaceae were sampled (Figure 2) and were grown on PDA (Difco Laboratories) for 2–7 days. Strains were evaluated on detached leaves of *Arabidopsis thaliana* (Col-0 ecotype), with three biological replicates. Mock controls were inoculated with PDA plugs. Plants were grown in environmental chambers at 22°C, 50% humidity, with a photoperiod of 12 hours of light and 12 hours of dark to promote vegetative growth. Leaves from 4- to 5-week-old plants were removed to Petri plates lined with moist filter paper, inoculated with colonized 4 mm agar plugs taken from the colony margin, then incubated at 80% humidity to promote infection and lesion development. Leaves were photographed at 16, 24, 48, 72 and 96 hours post-inoculation. Lesion area and total leaf area in square millimeters was obtained from high-resolution digital images of infected leaves using ImageJ [80].

### *In vitro*, bromophenol blue indicator plate screening for oxalic acid

The protocol of Godoy *et al.* [81], who demonstrated that oxalic acid-deficient mutants of *S. sclerotiorum* do not produce a color change, was followed with one important modification. Because some strains grew very slowly and others rapidly, inoculum was standardized as a homogenate with 8–10×10^3^ mycelial fragments per ml. Each indicator plate was spread with 100 µL of inoculum, with three replicates for each strain. Plates were photographed every 24 hours until the plates changed from purple to yellow, or until the plates became overgrown. The color change indicated acid production, which we have taken as a proxy for relative oxalic acid concentration, consistent with results of Godoy *et al.* [81], Dickman and Mitra [43], and Germeier *et al.* [44].

### *In planta* oxalic acid pathway-related gene expression assay

*A. thaliana* plants were grown and inoculated with colonized agar plugs as described in the virulence testing section. The first trial included the reference necrotrophic generalists, *B. cinerea* (B05.10) and *S. sclerotiorum* (1980), a host specialist of unknown trophic type, *S. glacialis* (LMK743), and the biotrophic specialists, *M. scirpicola* (LMK735) and *M. duriaeana* (LMK746). The second trial included the same two reference necrotrophic generalists, the necrotrophic specialists, *B. tulipae* (LMK76), *Monilinia aucuparieae* (LMK738), and *M. fructicola* (LMK125), and the biotrophic specialist, *Myriosclerotinia curreyana* (LMK733). All isolates selected for this study were medium- to fast-growing (3 to 5 days to cover a 100 mm Petri plate). Leaves were sampled, then frozen in liquid nitrogen, at 1, 2, 4, 8, 12, 24, 48 and 72 hours post-inoculation. Three leaves from one plant were sampled at each time point as biological replicates for each strain. Total RNA was extracted using the RNeasy Mini kit (Qiagen), according to the manufacturer's recommendations. Four micrograms of total RNA was treated with DNase I (Invitrogen, Carlsbad, CA) prior to reverse transcription to avoid any genomic DNA contamination. Reverse transcription was performed using the SuperScript First-Strand Synthesis System (Invitrogen).

Primers and TaqMan probes for qRT-PCR were designed using Beacon Designer 7.0 (Premier Biosoft International, Palo Alto, USA). The highest scoring primer and probe set for each target was selected. All probes were 5′ labeled with FAM (6-carboxyfluorescein) as a fluorescent reporter and 3′ labeled with Iowa Black FQ as a quencher. qRT-PCR was performed to amplify the pathogenicity-related genes (*oah* and *pac1*) and the reference gene (*act*) for using each primer and probe set detailed in Table S6. The genes, *oah* and *pac1*, were chosen based on the importance of oxalic acid in modulating pH throughout plant infection, unlike CWDEs that are produced early in infection. We were also able to amplify the oxalic acid-related genes from almost all strains tested, while genes encoding CWDEs did not amplify consistently (Figure 1B).

qRT-PCR reactions were based on the 5′ nuclease assay. The 25 µl reactions contained 1X PCR buffer [20 mM Tris-HCl (pH 8.4) and 50 mM KCl], 1.8 mM MgCl_2_, 200 mM dNTPs, 400 nM of each forward and reverse primer, 200 nM of TaqMan probe, 0.625 U Platinum *Taq* DNA polymerase (Invitrogen), and 30 nM of ROX reference dye (Stratagene, La Jolla, USA). For each sample, 5′ nuclease reactions were replicated three times, and included three no-reverse transcription controls to test for the presence of genomic DNA, and three no-template controls that contained nuclease free water (Integrated DNA Technologies, Coralville, USA) instead of template. All experimental samples were tested against standards of known concentration. These standards were produced for each primer and probe set using a 10-fold dilution series that ranged from 10^0^ (i.e. from 1 to 9 molecules) to 10^7^ molecules of linearized plasmid. The quantities of DNA in the highest concentration standards were determined using a NanoDrop 1000 spectrophotometer (Thermo Fisher Scientific, Waltham, USA). Thermal cycling was carried out in an Mx3000P qPCR System (Stratagene) with the following parameters: 95°C for 5 min, and 40 cycles of 95°C for 15 s followed by 60°C for 1 min.

qRT-PCR results were analysed using MxPro QPCR software (Stratagene) to obtain gene copy number. Pathogenicity-related gene expression was calculated relative to the transcripts levels of the constitutively expressed housekeeping gene, *act*, [29], [82] using the formula 2^−ΔCt^ = 2^−(Ctpathgene−Ct*act*)^ [83]. Gene expression values that were lower than the experimental threshold, as set by the concentration of the most dilute standard, were not included in all analyses. Tests for homogeneity of variance were conducted using JMP 8.0 (SAS Institute Inc., Cary, NC, 1989–2007) after all copy number values were transformed. A split plot factorial repeated measures analysis was also performed in JMP 8.0 to test for differences between species and across time. The normalized pathogenicity-related gene expression was also calculated from RNAs from pure fungal cultures to determine whether expression of these genes was induced *in planta*, or was expressed constitutively.

## Supporting Information

Figure S1 **Gene tree topology inferred from** ***oah*** **sequence data using Bayesian inference.** Thick branches represent well-supported nodes with >90% support from 1000 maximum likelihood bootstrapped pseudoreplicates and >0.95 posterior probabilities.
(TIF)

Figure S2 **Gene tree topology inferred from** ***pac1*** **sequence data using Bayesian inference.** Thick branches represent well-supported nodes with >90% support from 1000 maximum likelihood bootstrapped pseudoreplicates and >0.95 posterior probabilities.
(TIF)

Figure S3 **Gene tree topology inferred from** ***asps*** **sequence data using Bayesian inference.** Thick branches represent well-supported nodes with >90% support from 1000 maximum likelihood bootstrapped pseudoreplicates and >0.95 posterior probabilities.
(TIF)

Figure S4 **Gene tree topology inferred from** ***pg1*** **sequence data using Bayesian inference.** Thick branches represent well-supported nodes with >90% support from 1000 maximum likelihood bootstrapped pseudoreplicates and >0.95 posterior probabilities.
(TIF)

Figure S5 **Gene tree topology inferred from** ***pg3*** **sequence data using Bayesian inference.** Thick branches represent well-supported nodes with >90% support from 1000 maximum likelihood bootstrapped pseudoreplicates and >0.95 posterior probabilities.
(TIF)

Figure S6 **Gene tree topology inferred from** ***pg5*** **sequence data using Bayesian inference.** Thick branches represent well-supported nodes with >90% support from 1000 maximum likelihood bootstrapped pseudoreplicates and >0.95 posterior probabilities.
(TIF)

Figure S7 **Gene tree topology inferred from** ***pg6*** **sequence data using Bayesian inference.** Thick branches represent well-supported nodes with >90% support from 1000 maximum likelihood bootstrapped pseudoreplicates and >0.95 posterior probabilities.
(TIF)

Figure S8 **Gene tree topology inferred from** ***acp1*** **sequence data using Bayesian inference.** Thick branches represent well-supported nodes with >90% support from 1000 maximum likelihood bootstrapped pseudoreplicates and >0.95 posterior probabilities.
(TIF)

Table S1 Site-specific likelihood analyses: Log likelihood (lnL) values, number of parameters (np), and parameter estimates for eight pathogenicity-related genes and two housekeeping genes.
(DOC)

Table S2 Branch-specific likelihood analyses: Log likelihood (lnL) values, number of parameters (np), and parameter estimates for six pathogenicity-related genes and two housekeeping genes. The null model fixes the *d*N/*d*S ratio across all lineages in the phylogeny, while the alternative model allows for a different *d*N/*d*S value for the foreground branch(es).
(DOC)

Table S3 Host and origin of strains examined in this study.
(DOC)

Table S4 Primer sets for amplification of housekeeping and pathogenicity-related genes in the Sclerotiniaceae.
(DOC)

Table S5 Supporting information on loci used in phylogenetic analyses of housekeeping and pathogenicity-related loci.
(DOC)

Table S6 Primer/probe sets for quantitative PCR of 2 pathogenicity-related loci (*oah* and *pac1*) and a reference gene (*actin*).
(DOC)
