# Supplementary figures and images for: Evidence for a Common Toolbox Based on Necrotrophy in a Fungal Lineage Spanning Necrotrophs, Biotrophs, Endophytes, Host Generalists and Specialists
Source: PLoS One. 2012 Jan 11;7(1):e29943. doi: 10.1371/journal.pone.0029943 (PMC3256194; doi:10.1371/journal.pone.0029943)

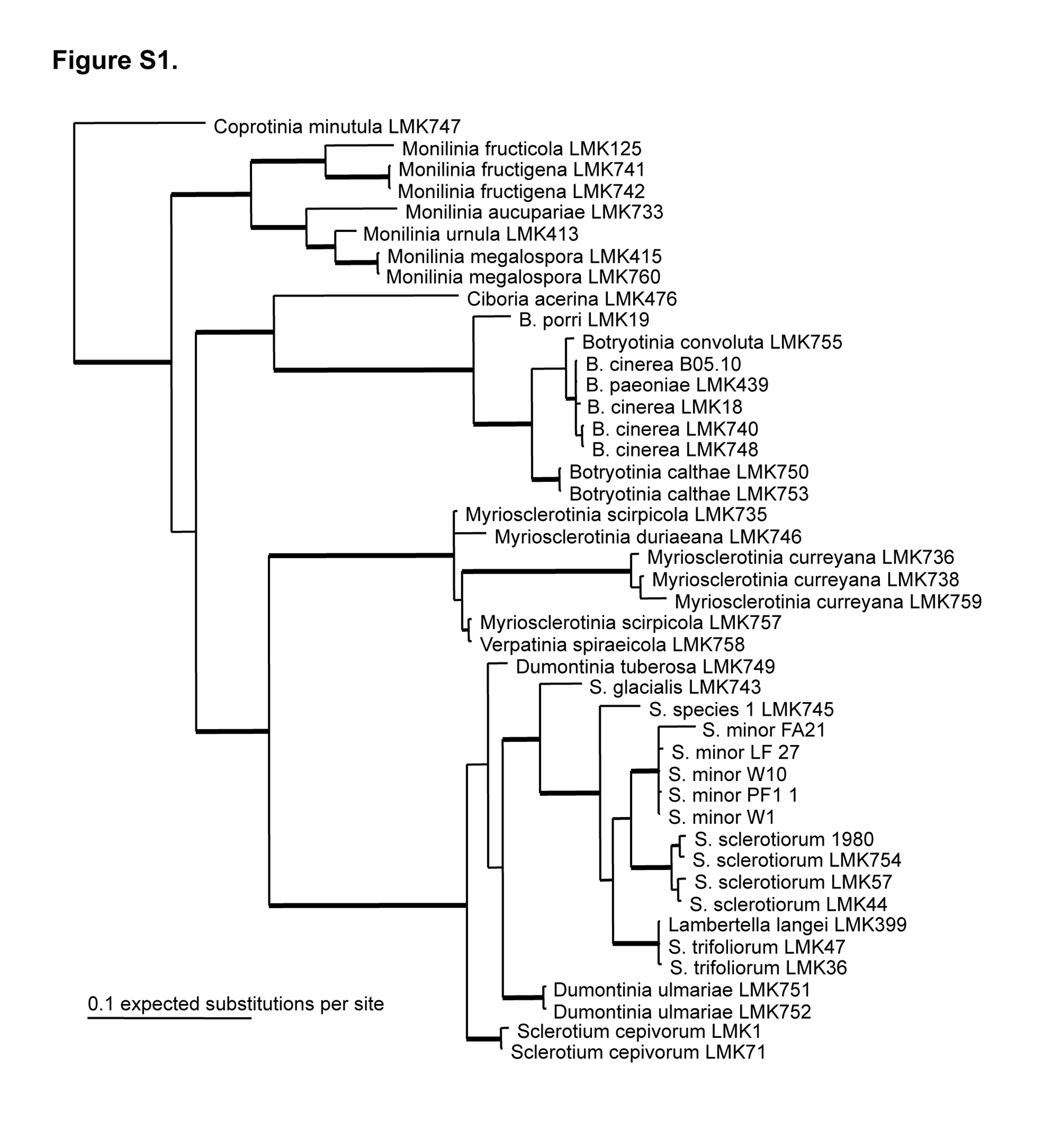

Supplement: Figure S1 — Gene tree topology inferred from oah sequence data using Bayesian inference. Thick branches represent well-supported nodes with >90% support from 1000 maximum likelihood bootstrapped pseudoreplicates and >0.95 posterior probabilities. (TIF) [file pone.0029943.s001.tif]

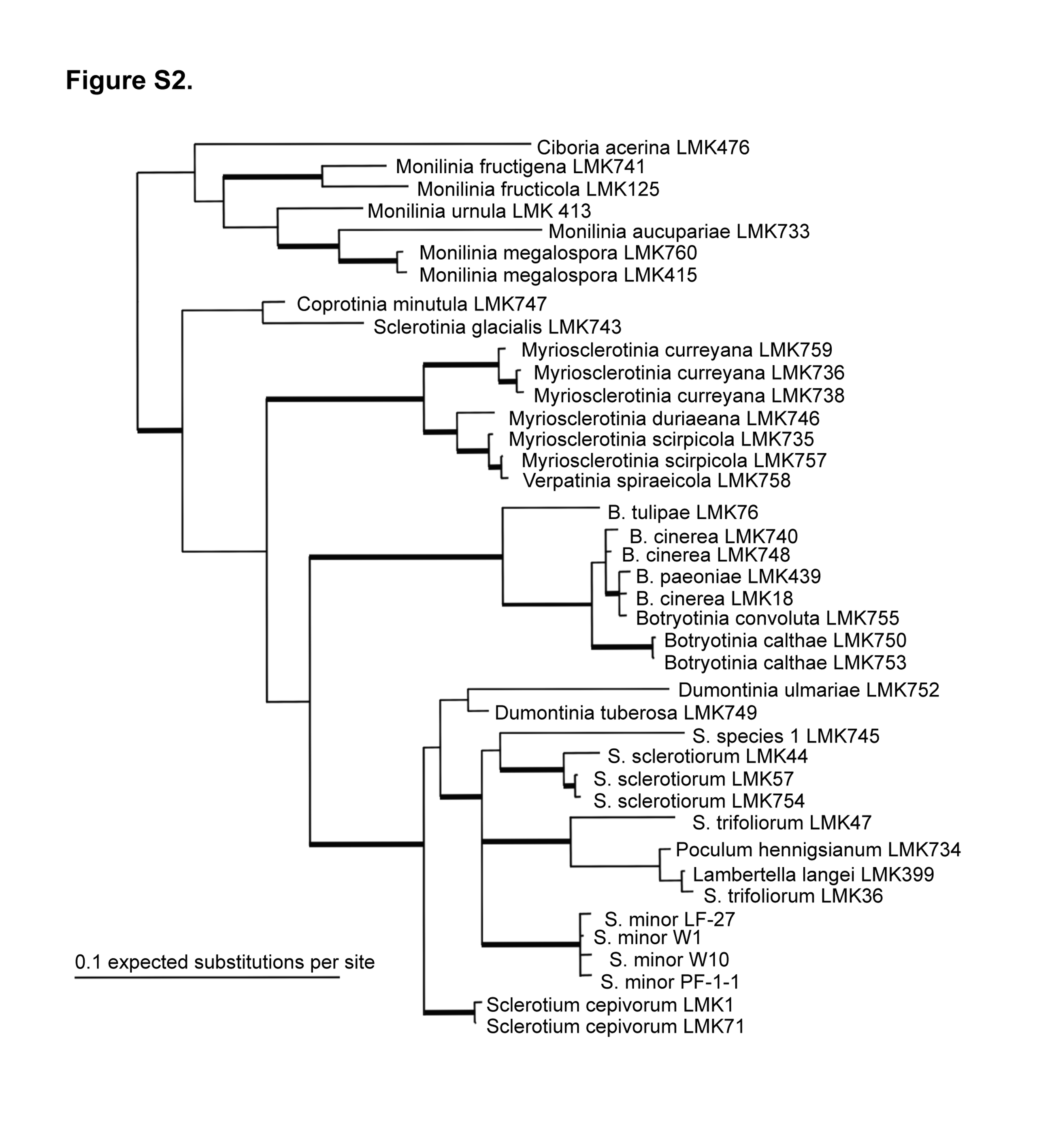

Supplement: Figure S2 — Gene tree topology inferred from pac1 sequence data using Bayesian inference. Thick branches represent well-supported nodes with >90% support from 1000 maximum likelihood bootstrapped pseudoreplicates and >0.95 posterior probabilities. (TIF) [file pone.0029943.s002.tif]

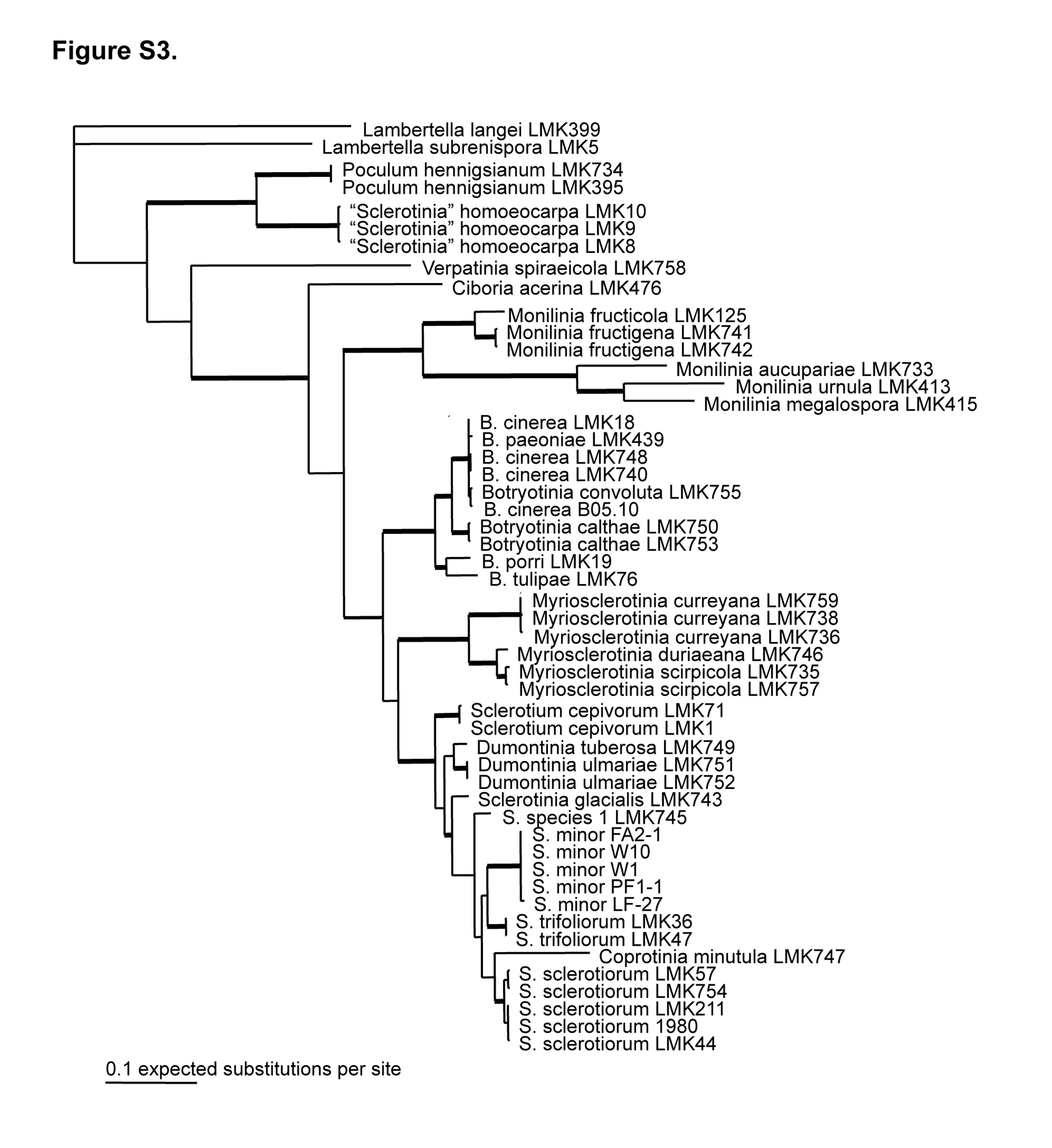

Supplement: Figure S3 — Gene tree topology inferred from asps sequence data using Bayesian inference. Thick branches represent well-supported nodes with >90% support from 1000 maximum likelihood bootstrapped pseudoreplicates and >0.95 posterior probabilities. (TIF) [file pone.0029943.s003.tif]

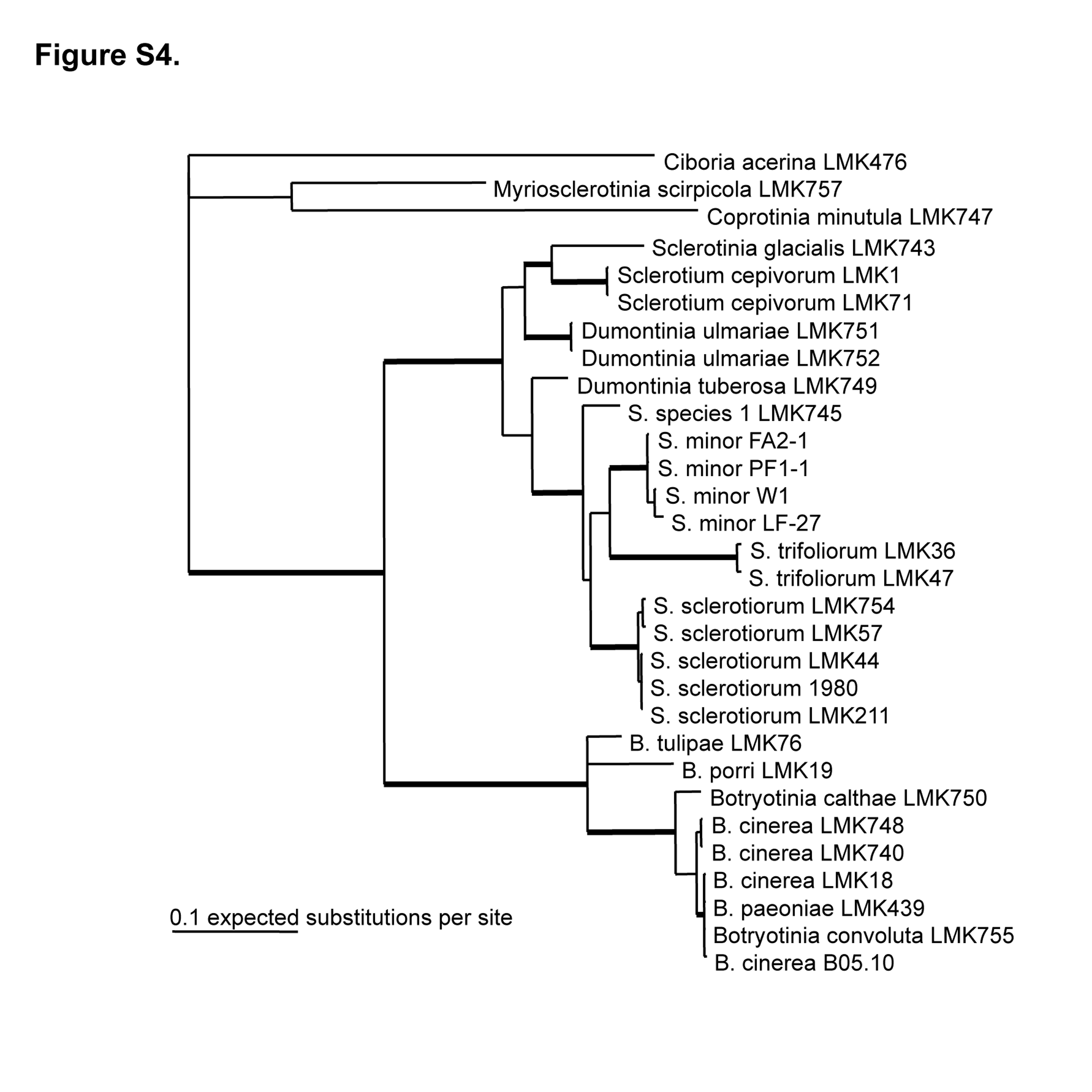

Supplement: Figure S4 — Gene tree topology inferred from pg1 sequence data using Bayesian inference. Thick branches represent well-supported nodes with >90% support from 1000 maximum likelihood bootstrapped pseudoreplicates and >0.95 posterior probabilities. (TIF) [file pone.0029943.s004.tif]

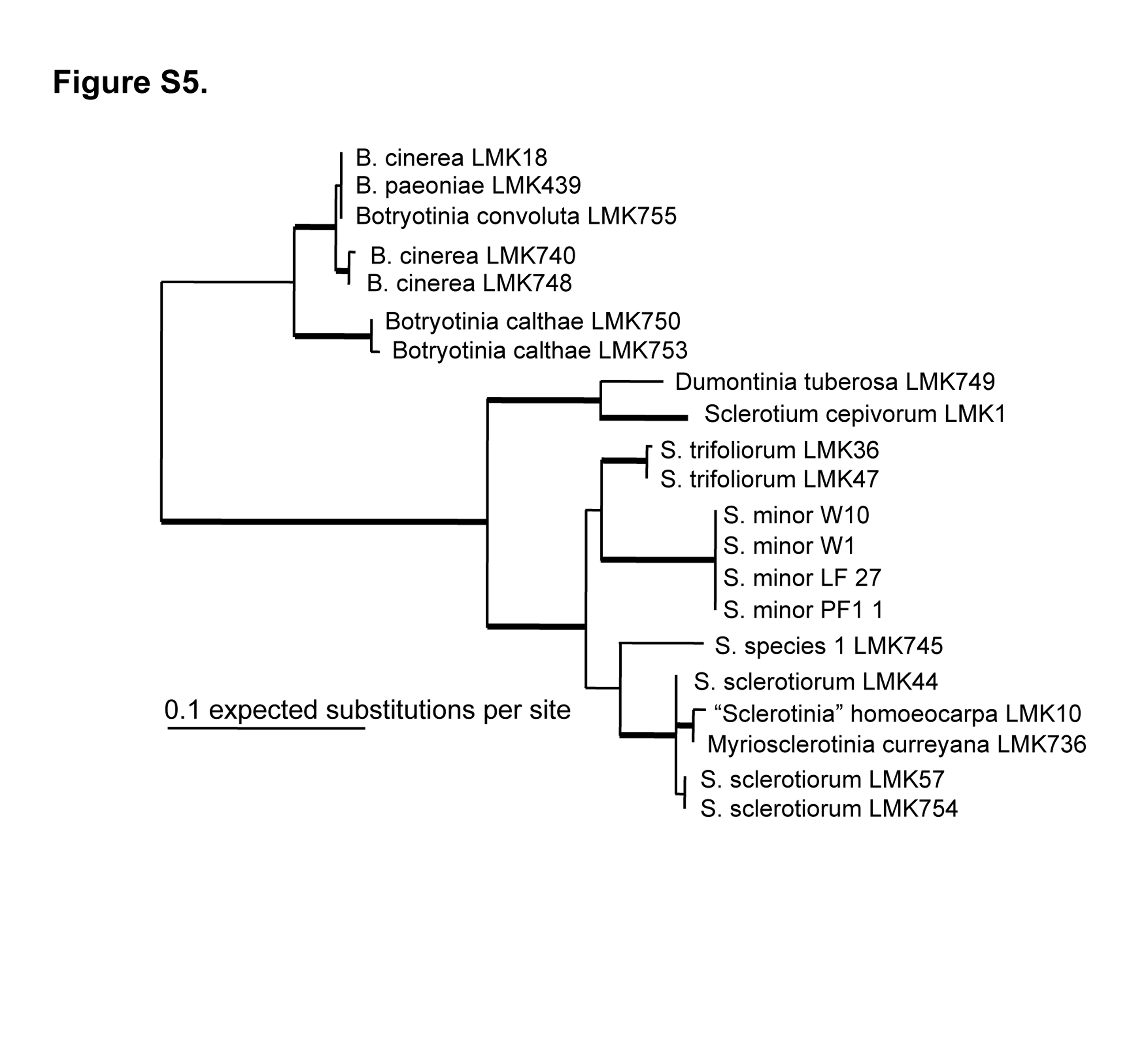

Supplement: Figure S5 — Gene tree topology inferred from pg3 sequence data using Bayesian inference. Thick branches represent well-supported nodes with >90% support from 1000 maximum likelihood bootstrapped pseudoreplicates and >0.95 posterior probabilities. (TIF) [file pone.0029943.s005.tif]

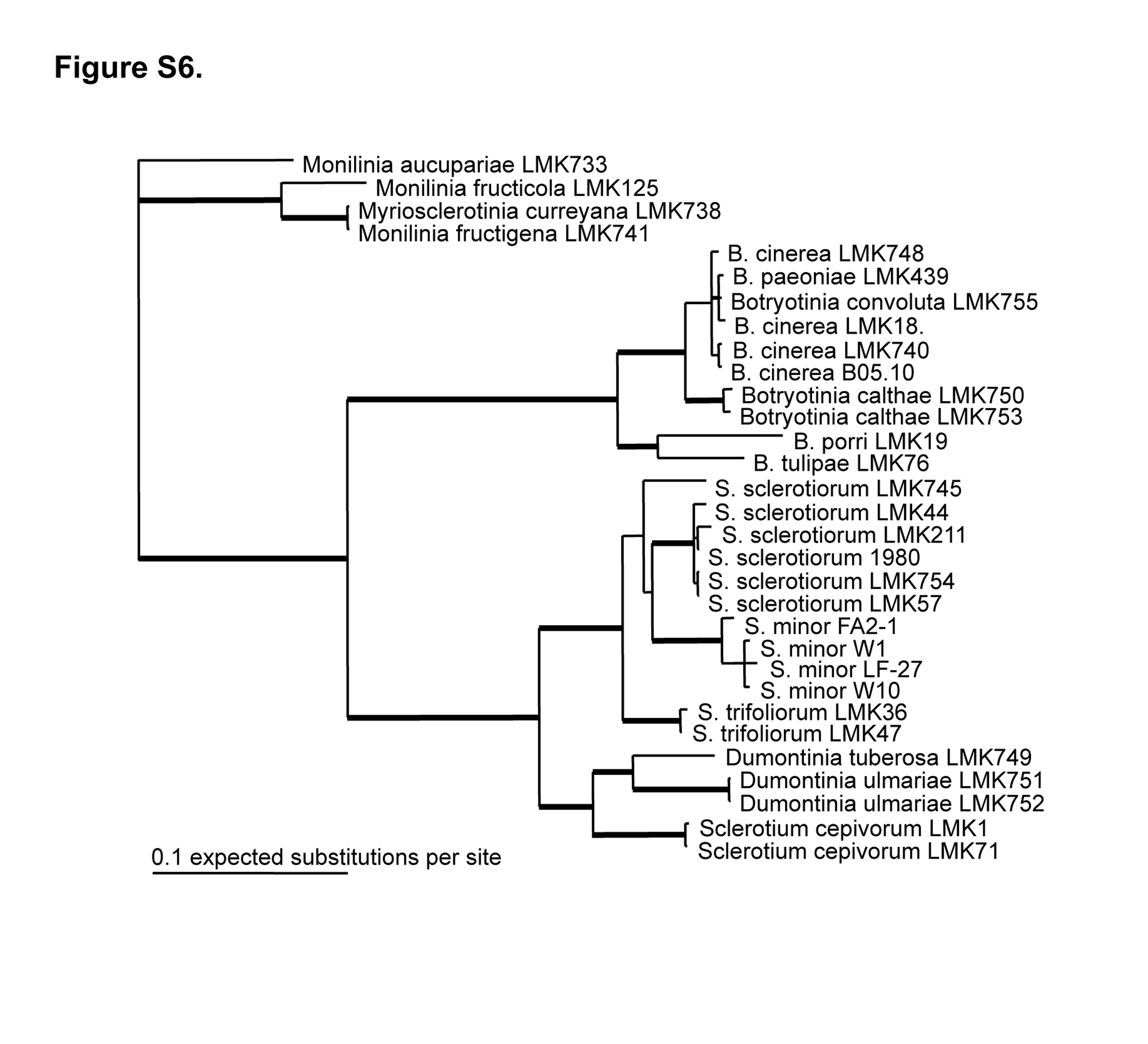

Supplement: Figure S6 — Gene tree topology inferred from pg5 sequence data using Bayesian inference. Thick branches represent well-supported nodes with >90% support from 1000 maximum likelihood bootstrapped pseudoreplicates and >0.95 posterior probabilities. (TIF) [file pone.0029943.s006.tif]

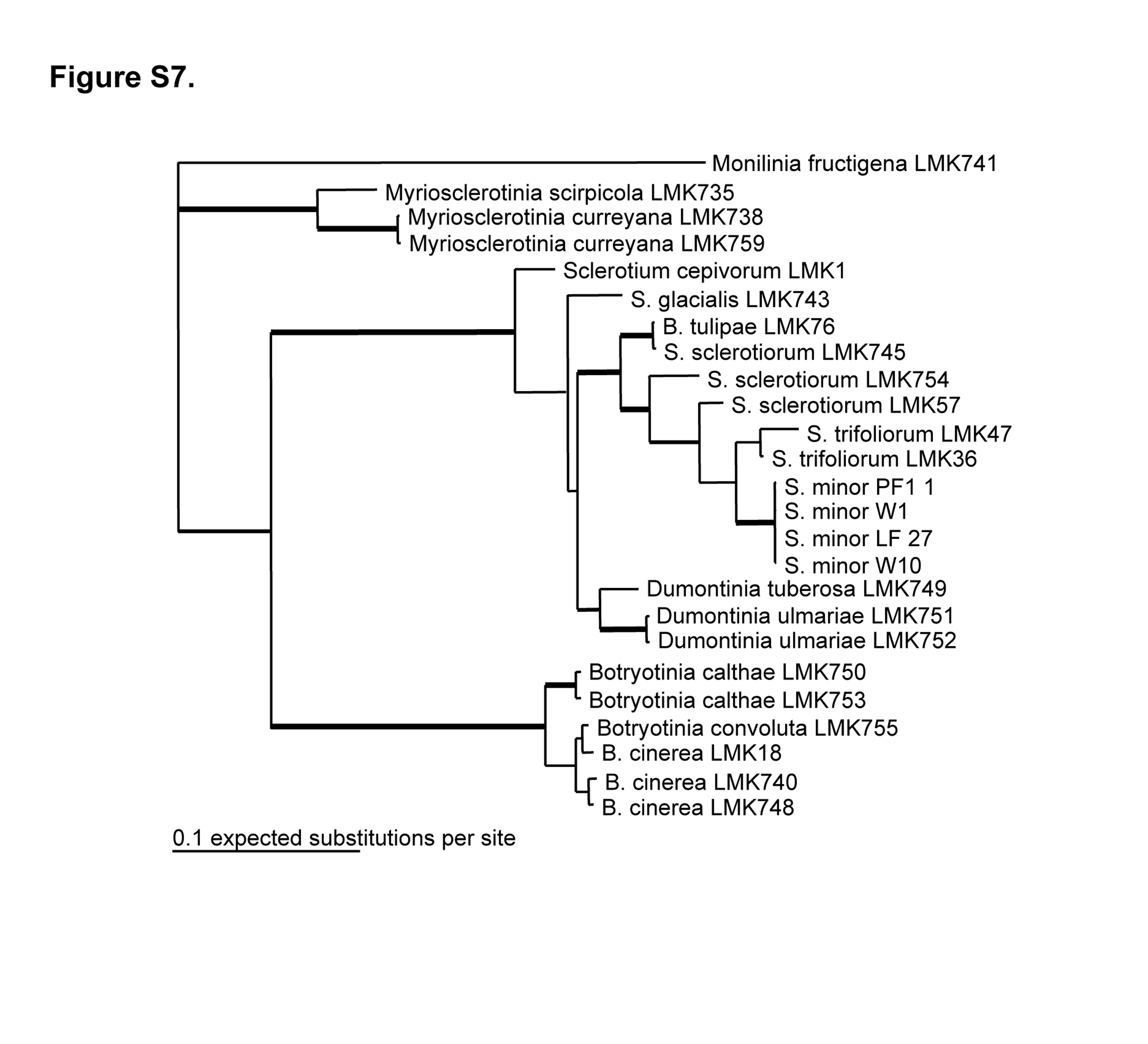

Supplement: Figure S7 — Gene tree topology inferred from pg6 sequence data using Bayesian inference. Thick branches represent well-supported nodes with >90% support from 1000 maximum likelihood bootstrapped pseudoreplicates and >0.95 posterior probabilities. (TIF) [file pone.0029943.s007.tif]

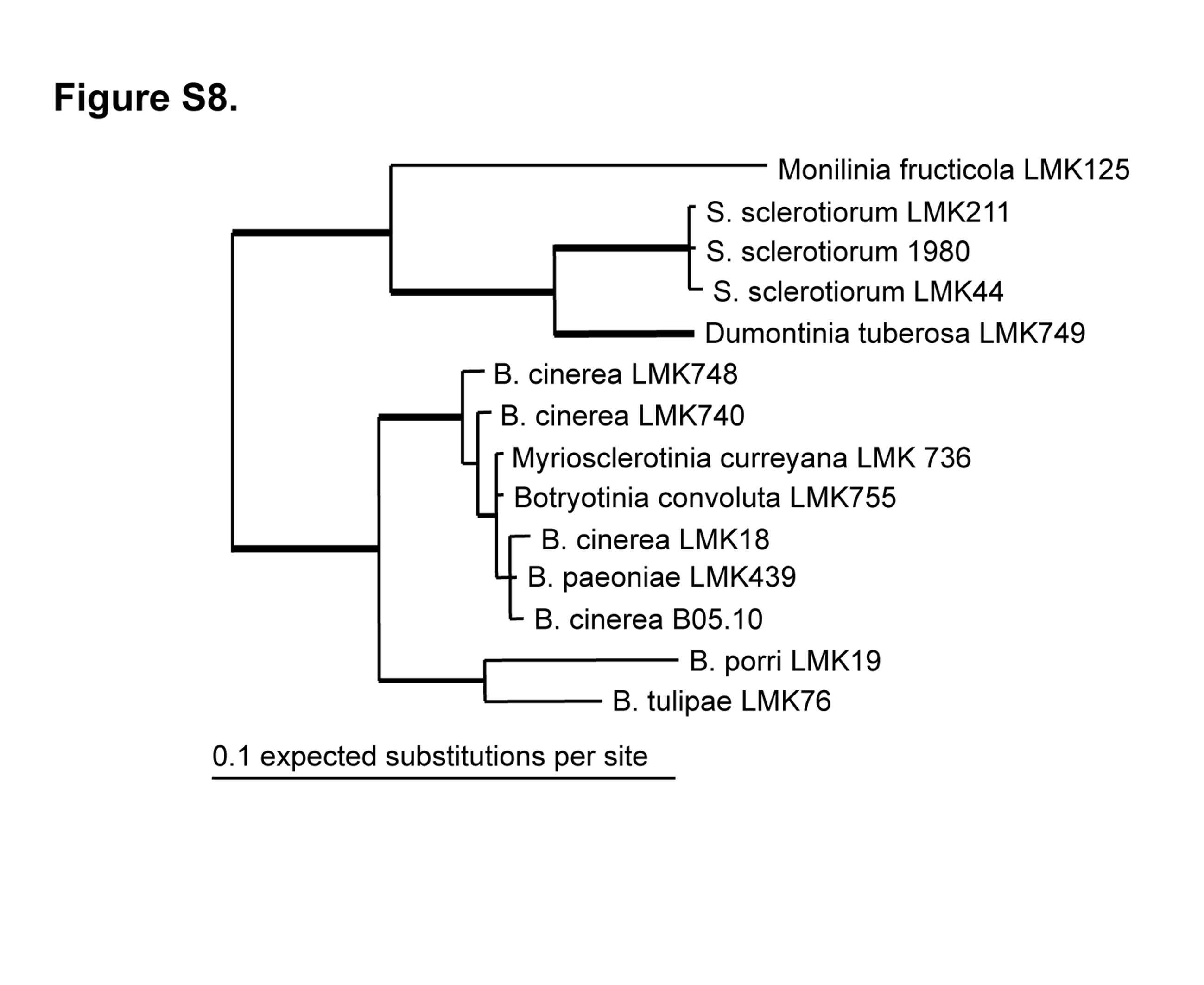

Supplement: Figure S8 — Gene tree topology inferred from acp1 sequence data using Bayesian inference. Thick branches represent well-supported nodes with >90% support from 1000 maximum likelihood bootstrapped pseudoreplicates and >0.95 posterior probabilities. (TIF) [file pone.0029943.s008.tif]
