# Supplementary material for: Evidence for a Common Toolbox Based on Necrotrophy in a Fungal Lineage Spanning Necrotrophs, Biotrophs, Endophytes, Host Generalists and Specialists
Source: PLoS One. 2012 Jan 11;7(1):e29943. doi: 10.1371/journal.pone.0029943 (PMC3256194; doi:10.1371/journal.pone.0029943)
Supplement: Table S1 — Site-specific likelihood analyses: Log likelihood (lnL) values, number of parameters (np), and parameter estimates for eight pathogenicity-related genes and two housekeeping genes. (DOC) [file pone.0029943.s009.doc]

**Table S1. Site-specific likelihood analyses: Log likelihood (lnL) values, number of parameters (np), and parameter estimates for eight pathogenicity-related genes and two housekeeping genes.**

| **Gene** | **Model code** | **lnL** | **Estimates of parameters** | **np** | **Positively selected sites** |
| --- | --- | --- | --- | --- | --- |
| *acp1* | M0 | -2329.500691 | ω=0.15086 | 28 | None |
|  | M1a | -2282.941525 | ω_0_=0.03466, (ω_1_=1), p_0_=0.81356, (p_1_=0.18644) ^a^ | 29 | Not allowed |
|  | M2a | -2271.811966 | ω_0_=0.03388, (ω_1_=1), ω_2_=15.83149, p_0_=0.80537, p_1_=0.19006, (p_2_=0.00457) | 31 | **204N ^c^** |
|  | M3 | -2266.762520 | ω_0_=0.00588, ω_1_=0.52595, ω_2_=13.26297, p_0_=0.70237, p_1_=0.29300, (p_2_=0.00463) | 32 | **204N** |
|  | M7 | -2278.428308 | p=0.11773, q=0.55569 ^b^ | 29 | Not allowed |
|  | M8 | -2266.884978 | p_0_=0.99539, (p_1_=0.00461), p=0.13710, q=0.68939, ω=13.49767 | 31 | **204N** |
| *asps* | M0 | -8919.048719 | ω=0.15994 | 70 | None |
|  | M1a | -8532.681216 | ω_0_=0.04504, (ω_1_=1), p_0_=0.78683, (p_1_=0.21317) | 71 | Not allowed |
|  | M2a | -8532.681136 | ω_0_=0.04504, (ω_1_=1), ω_2_=1, p_0_=0.78683, p_1_=0.12508, (p_2_=0.08809) | 73 | None |
|  | M3 | -8485.202596 | ω_0_=0.01229, ω_1_=0.26634, ω_2_=1.04569, p_0_=0.6373, p_1_=0.23023, (p_2_=0.13104) | 74 | **16Q,** **26V,** 53V, 68A, **69G, 72V, 76T,** 79Q, 87V, 126A, **134T,** **179Q, 183Q,** 206T, **222Q,** 233A, **252S,** 268T, 276Q, 325N, **327Q, 332Q, 342T** |
|  | M7 | -8489.185656 | p= 0.15479, q=0.63116 | 71 | Not allowed |
|  | M8 | -8479.427215 | p_0_= 0.97042, (p_1_=0.02958), p=0.18149, q=0.94374, ω=1.92177 | 73 | 134T |
| *oah* | M0 | -3897.107810 | ω = 0.04522 | 65 | None |
|  | M1a | -3872.870242 | ω_0_ = 0.02472, (ω_1_=1), p_0_=0.94594, (p_1_=0.05406) | 66 | Not allowed |
|  | M2a | -3872.870242 | ω_0_ = 0.02472, (ω_1_=1), ω_2_=22.69859, p_0_=0.94594, p_1_=0.05406, (p_2_=0) | 68 | None |
|  | M3 | -3843.324994 | ω_0_ = 0, ω_1_=0.00109, ω_2_=0.25933, p_0_=0.02468, p_1_=0.79274, (p_2_=0.18258) | 69 | None |
|  | M7 | -3844.368561 | p= 0.08855, q=1.59665 | 66 | Not allowed |
|  | M8 | -3844.366025 | p_0_= 0.99999, (p_1_ = 0.00001), p=0.08855, q=1.59665, ω=3.65341 | 68 | None |
| *pac1* | M0 | -6132.517449 | ω = 0.09911 | 50 | None |
|  | M1a | -6083.830891 | ω_0_ = 0.06334, (ω_1_=1), p_0_=0.92087, (p_1_= 0.07913) | 51 | Not allowed |
|  | M2a | -6083.830891 | ω_0_ = 0.06334, (ω_1_=1), ω_2_=1, p_0_= 0.92087, p_1_= 0.04642, (p_2_=0.03271) | 53 | None |
|  | M3 | -6058.322109 | ω_0_=0.02363, ω_1_=0.36072, ω_2_= 3.42959, p_0_= 0.75525, p_1_= 0.24209, (p_2_=0.00266) | 54 | **394L** |
|  | M7 | -6061.749994 | p= 0.26858, q=2.08443 | 51 | Not allowed |
|  | M8 | -6058.318444 | p_0_= 0.99741, (p_1_ = 0.00259), p=0.28660, q=2.32961, ω= 3.48498 | 53 | 394L |

| *pg1* | M0 | -2569.946193 | ω = 0.09124 | 26 | None |
| --- | --- | --- | --- | --- | --- |
|  | M1a | -2550.868370 | ω_0_ = 0.05132, (ω_1_=1), p_0_=0.91679, (p_1_= 0.08321) | 27 | Not allowed |
|  | M2a | -2550.868370 | ω_0_ = 0.05132, (ω_1_=1), ω_2_=1, p_0_= 0.91679, p_1_= 0.04720, (p_2_=0.03601) | 29 | None |
|  | M3 | -2546.617433 | ω_0_ = 0, ω_1_= 0.20783, ω_2_= 1.05957, p_0_= 0.59456, p_1_= 0.37339, (p_2_=0.03205) | 30 | None |
|  | M7 | -2547.035662 | p= 0.22001, q=1.73313 | 27 | Not allowed |
|  | M8 | -2546.928491 | p_0_= 0.98382, (p_1_ = 0.01618), p=0.25812, q=2.37659, ω= 1.13113 | 29 | None |
| *pg3* | M0 | -4020.680055 | ω=0.23324 | 38 | None |
|  | M1a | -3954.533844 | ω_0_ = 0.03310, (ω_1_=1), p_0_=0.73454, (p_1_= 0.26546) | 39 | Not allowed |
|  | M2a | -3952.276803 | ω_0_ = 0.03534, (ω_1_=1), ω_2_= 5.84296, p_0_= 0.73588, p_1_= 0.25309, (p_2_=0.01103) | 41 | None |
|  | M3 | -3951.572575 | ω_0_ = 0, ω_1_= 0.56686, ω_2_= 2.94474, p_0_= 0.61027, p_1_= 0.34657, (p_2_=0.04316) | 42 | 136L, 258N |
|  | M7 | -3954.840538 | p=0.07670, q=0.19813 | 39 | Not allowed |
|  | M8 | -3951.653571 | p_0_= 0.98188, (p_1_= 0.01812), p=0.10409, q= 0.30759, ω= 4.51623 | 41 | 136L, 258N |
| *pg5* | M0 | -4023.732747 | ω=0.20104 | 38 | None |
|  | M1a | -3901.518983 | ω_0_ = 0.03539, (ω_1_=1), p_0_=0.78004, (p_1_= 0.21996) | 39 | Not allowed |
|  | M2a | -3898.046275 | ω_0_ = 0.03759, (ω_1_=1), ω_2_= 3.41761, p_0_= 0.77964, p_1_= 0.19561, (p_2_=0.02475) | 41 | None |
|  | M3 | -3893.565163 | ω_0_ =0.01817, ω_1_=0.52733, ω_2_= 2.41802, p_0_= 0.70156, p_1_= 0.24457, (p_2_=0.05388) | 42 | 47S, 89K, 150S, 216D, 224T, **279S**, 293G |
|  | M7 | -3901.506352 | p=0.10950, q=0.39519 | 39 | Not allowed |
|  | M8 | -3894.069947 | p_0_= 0.95627, (p_1_= 0.04373), p=0.15513, q= 0.76968, ω= 2.61653 | 41 | 279S |
| *pg6* | M0 | -4062.973592 | ω=0.14767 | 46 | None |
|  | M1a | -3980.295815 | ω_0_ = 0.04024, (ω_1_=1), p_0_=0.82864, (p_1_= 0.17136) | 47 | Not allowed |
|  | M2a | -3980.295815 | ω_0_ = 0.04024, (ω_1_=1), ω_2_=1, p_0_= 0.82863, p_1_= 0.1112, (p_2_= 0.06015) | 49 | None |
|  | M3 | -3976.675216 | ω_0_ = 0.01753, ω_1_=0.41217, ω_2_= 1.46976, p_0_= 0.72098, p_1_=0.22384, (p_2_=0.05518) | 50 | 312S, 317E |
|  | M7 | -3978.435472 | p= 0.13288, q=0.62674 | 47 | Not allowed |
|  | M8 | -3976.906914 | p_0_= 0.94881, (p_1_ = 0.05119), p=0.19184, q=1.39421, ω= 1.45142 | 49 | None |
| *g3pdh* | M0 | -3414.627492 | ω = 0.07805 | 86 | None |
|  | M1a | -3348.114730 | ω_0_ = 0.02895, (ω_1_=1), p_0_=0.91916, (p_1_= 0.08084) | 87 | Not allowed |
|  | M2a | -3348.114730 | ω_0_ = 0.02895, (ω_1_=1), ω_2_=1, p_0_= 0.91916, p_1_=0.05604, (p_2_= 0.02480) | 89 | None |
|  | M3 | -3337.236457 | ω_0_ = 0, ω_1_= 0.17627, ω_2_= 0.91168, p_0_= 0.73802, p_1_= 0.20251, (p_2_=0.05947) | 90 | None |
|  | M7 | -3339.145488 | p= 0.07854, q=0.73132 | 87 | Not allowed |
|  | M8 | -3339.145248 | p_0_= 1.00000, (p_1_ = 0), p=0.07854, q=0.73133, ω= 3.85934 | 89 | None |
| *hsp60* | M0 | -5629.645740 | ω = 0.01682 | 104 | None |
|  | M1a | -5628.478560 | ω_0_ = 0.01553, (ω_1_=1), p_0_=0.99404, (p_1_= 0.00596) | 105 | Not allowed |
|  | M2a | -5628.478560 | ω_0_ = 0.01553, (ω_1_=1), ω_2_=20.74179, p_0_=0.99404, p_1_=0.00596, (p_2_= 0) | 107 | None |
|  | M3 | -5604.381399 | ω_0_ = 0.00002, ω_1_=0.00633, ω_2_= 0.10963, p_0_= 0.60541, p_1_=0.24436, (p_2_=0.15022) | 108 | None |
|  | M7 | -5604.523767 | p= 0.10131, q=4.50777 | 105 | Not allowed |
|  | M8 | -5604.524054 | p_0_= 1.00000, (p_1_ = 0), p=0.10131, q=4.50776, ω= 2.28912 | 107 | None |

^a^ Proportions that are not free parameters are in parentheses.

^b^ The parameters p and q describe the shape of the beta distribution of ω.

^c^ Amino acid sites inferred to be under positive selection with posterior probability (PP) > 95%. Sites with PP > 99% are in bold.
