# Supplementary material for: Evidence for a Common Toolbox Based on Necrotrophy in a Fungal Lineage Spanning Necrotrophs, Biotrophs, Endophytes, Host Generalists and Specialists
Source: PLoS One. 2012 Jan 11;7(1):e29943. doi: 10.1371/journal.pone.0029943 (PMC3256194; doi:10.1371/journal.pone.0029943)
Supplement: Table S2 — Branch-specific likelihood analyses: Log likelihood (lnL) values, number of parameters (np), and parameter estimates for six pathogenicity-related genes and two housekeeping genes. The null model fixes the dN/dS ratio across all lineages in the phylogeny, while the alternative model allows for a different dN/dS value for the foreground branch(es). (DOC) [file pone.0029943.s010.doc]

**Table S2.** **Branch-specific likelihood analyses: Log likelihood (lnL) values, number of parameters (np), and parameter estimates for six pathogenicity-related genes and two housekeeping genes.** The null model fixes the *d*N/*d*S ratio across all lineages in the phylogeny, while the alternative model allows for a different *d*N/*d*S value for the foreground branch (set to the branch that separates the *Myriosclerotinia* clade from all others).

| **Gene** | **Model code** | **lnL** | **Estimates of parameters** | **np** | **Positively selected sites** |
| --- | --- | --- | --- | --- | --- |
| *acp1* | Null | -2282.941522 | p_0_ = 0.66362 , p_1_ = 0.15208, (p_2_ + p_3_) = 0.18430, ω_0_ = 0.03466 | 30 | Not allowed |
|  | Alternative | -2282.941522 | p_0_ = 0.64924 , p_1_ = 0.14878, (p_2_ + p_3_) = 0.20198, ω_2_ = 1.28083 | 31 | None |
| *asps* | Null | -8532.680653 | p_0_ = 0.78683, p_1_ = 0.21317, (p_2_ + p_3_) = 0, ω_0_ = 0.04504 | 72 | Not allowed |
|  | Alternative | -8532.680653 | p_0_ = 0.78683, p_1_ = 0.21317, (p_2_ + p_3_) = 0, ω_2_ = 1.0000 | 73 | None |
| *oah* | Null | -3872.870242 | p_0_= 0 0.94594, p_1_ = 0.05406, (p_2_ + p_3_)=0, ω_0_ = 0.02472 | 67 | Not allowed |
|  | Alternative | -3872.870242 | p_0_ =0.94593, p_1_ = 0.05407, (p_2_ + p_3_) = 0, ω_2_ = 1.00000 | 68 | None |
| *pac1* | Null | -6083.830891 | p_0_= 0.91503, p_1_ = 0.07859, (p_2_ + p_3_)=0.00638, ω_0_ = 0.06294 | 52 | Not allowed |
|  | Alternative | -6083.830891 | p_0_ = 0.92087, p_1_ = 0.07913, (p_2_ + p_3_) = 0, ω_2_ = 18.43177 | 53 | None |
| *pg1* | Null | -2542.400238 | p_0_= 0.80911, p_1_ = 0.06425, (p_2_ + p_3_)=0.12664, ω_0_ = 0.03234 | 28 | Not allowed |
|  | Alternative | -2542.398874 | p_0_ = 0.81125, p_1_ = 0.06430, (p_2_ + p_3_) = 0.12445, ω_2_ = 1.03110 | 29 | None |
| *pg6* | Null | -3980.295815 | p_0_=0.82864, p_1_ = 0.17136, (p_2_ + p_3_)=0, ω_0_ = 0.04024 | 48 | Not allowed |
|  | Alternative | -3977.667451 | p_0_ =0.82418, p_1_ = 0.16760, (p_2_ + p_3_)= 0.00822, ω_2_ = 999.000 | 49 | None |
| *g3pdh* | Null | -3348.111916 | p_0_=0.91609, p_1_ =0.08055, (p_2_ + p_3_)=0.00336, ω_0_ = 0.02888 | 88 | Not allowed |
|  | Alternative | -3348.111889 | p_0_=0.91613, p_1_ = 0.08055, (p_2_ + p_3_)=0.00332, ω_0_ = 1.01452 | 89 | None |
| *hsp60* | Null | -5628.478560 | p_0_=0.99404, p_1_ =0.00596, (p_2_ + p_3_)=0, ω_0_ = 0.01553 | 106 | Not allowed |
|  | Alternative | -5628.478560 | p_0_=0.99404, p_1_ = 0.00596, (p_2_ + p_3_)=0, ω_0_ = 0.01553 | 107 | None |
