# Supplementary material for: Evidence for a Common Toolbox Based on Necrotrophy in a Fungal Lineage Spanning Necrotrophs, Biotrophs, Endophytes, Host Generalists and Specialists
Source: PLoS One. 2012 Jan 11;7(1):e29943. doi: 10.1371/journal.pone.0029943 (PMC3256194; doi:10.1371/journal.pone.0029943)
Supplement: Table S3 — Host and origin of strains examined in this study. (DOC) [file pone.0029943.s011.doc]

# Table S3. Host and origin of strains examined in this study.

| **Species name** | **Isolate code** ^a^ | **Alternate code** ^b^ | **Host** | | **Origin** |
| --- | --- | --- | --- | --- | --- |
| *Botryotinia calthae* Hennebert | LMK750 | 2336.P | *Caltha palustris* | | Telemark, Norway |
| *Botryotinia calthae* | LMK753 | 2326.P | | *Caltha palustris* | Vestfold, Norway |
| *Botryotinia convoluta* (Drayton) Whetzel | LMK755 | 2441.P | *Iris pseudacorus* | | Telemark, Norway |
| *Botrytis cinerea* Pers.:Fr. | B05.10 ^c^ |  |  | | d |
| *Botrytis cinerea* | LMK740 | 1061 | *Rubus chamaemorus* | | Oppland, Norway |
| *Botrytis cinerea* | LMK748 | 1971.K | *Filipendula ulmaria* | | Buskerud, Norway |
| *Botrytis cinerea* | LMK18 | 1882.K | *Allium cepa* | | Ontario, Canada |
| *Botrytis paeoniae* Oud. | LMK439 | 1892.K | *Paeonia* sp. | |  |
| *Botrytis porri* Buchw. | LMK19 | 1883.K | *Allium sativum* | | Oregon, USA |
| *Botrytis tulipae* (Lib.) Lind | LMK76 | 1890.K | *Tulipa gesneriana* | | Ontario, Canada |
| *Ciboria acerina* Whetzel & Buchw. | LMK476 | 1854 | *Myrica gale* | | Buskerud, Norway |
| *Coprotinia minutula* Whetzel | LMK747 | 1916.P | *Coleoptera* dung | | Ontario, Canada |
| *Dumontinia tuberosa* (Hedw.) L.M. Kohn | LMK749 | 2271.P | *Anemone nemorosa* | | Olso, Norway |
| *Dumontinia ulmariae* (Velen.) A. Holst-Jensen | LMK751 | 2317.P | *Filipendula ulmaria* | | Buskerud, Norway |
| *Dumontinia ulmariae* | LMK752 | 2318.P | | *Filipendula ulmaria* | Buskerud, Norway |
| *Lambertella langei* T. Schumach. & S. Holøs | LMK399 | 681.1 | *Andromeda polyfolia* | | Hedmark, Norway |
| *Lambertella subrenispora* Korf & Zhuang | LMK5 | 1879.P = CUP-JA 3663 | *Aster ageratoides* | | Japan |
| *Monilinia aucupariae* (Ludw.) Whetzel | LMK733 | 410.P | *Sorbus aucuparia* | | Oppland, Norway |
| *Monilinia fructicola* (Winter) Honey | LMK125 | 783.K | *Prunus persica* | | Ontario, Canada |
| *Monilinia fructigena* Honey | LMK741 | 1079.K | *Prunus domesticus* | | Akershus, Norway |
| *Monilinia fructigena* | LMK742 | 1127.K | | *Malus pumila* | Olso, Norway |
| *Monilinia megalospora* (Woronin) Whetzel | LMK415 | 481.1 | *Vaccinium vitis-idea* | | Akershus, Norway |
| *Monilinia megalospora* | LMK760 | 619.1 | | *Vaccinium uliginosum* | Oppland, Norway |
| *Monilinia urnula* (Weinm.) Whetzel | LMK413 | 476.1 | *Vaccinium vitis-idea* | | Akershus, Norway |
| *Myriosclerotinia curreyana* (Berk.) Buchw. | LMK736 | 693.2 | | *Juncus filiformis* | Hedmark, Norway |
| *Myriosclerotinia curreyana* | LMK738 | 739.1 | *Juncus arcticus* | | Oppland, Norway |
| *Myriosclerotinia curreyana* | LMK759 | 2461 | *Juncus arcticus* | | Hedmark, Norway |
| *Myriosclerotinia duriaeana* (C. Tul. & Tul.) Buchw. | LMK746 | 1913.1 | *Carex aquatilis* | | Ontario, Canada |
| *Myriosclerotinia scirpicola* (Rehm) Buchw. | LMK735 | 647.2 | | *Scirpus maritimus* | Akershus, Norway |
| *Myriosclerotinia scirpicola* | LMK757 | 2443.P | *Scirpus lacustris* | | Telemark, Norway |
| *Poculum henningsianum* (Plöttn.) T. Schumach.  & L.M. Kohn | LMK395 | 603.1 | | *Carex juncellata* | Oslo, Norway |
| *Poculum henningsianum* | LMK734 | 608.P | *Carex rostrata* | | Oslo, Norway |
| *Sclerotinia glacialis* F. Graf & T. Schumach. | LMK743 | 1129 | *Ranunculus glacialis* | | Grisons, Switzerland |
| *“Sclerotinia” homoeocarpa* Bennett | LMK8 | 1880.S | | *Agrostis palustris* | Pennsylvania, USA |
| *“Sclerotinia” homoeocarpa* | LMK9 | 1833.P | | *Agrostis palustris* | Pennsylvania, USA |
| *“Sclerotinia” homoeocarpa* | LMK10 | 1881.P | *Agrostis palustris* | | Pennsylvania, USA |
| *Sclerotinia minor* Jagger | FA 2-1 |  | *Arachis hypogaea* | | North Carolina, USA |
| *Sclerotinia minor* | LF-27 |  | *Lactuca sativa* | | USA |
| *Sclerotinia minor* | PF 1-1 |  | | *Arachis hypogaea* | North Carolina, USA |
| *Sclerotinia minor* | W1 |  | *Cyperus esculentus* | | North Carolina, USA |
| *Sclerotinia minor* | W10 |  | *Oenothra laciniata* | | North Carolina, USA |
| *Sclerotinia sclerotiorum* | 1980 ^e^ | ATCC 18683 | *Phaesolus vulgaris* | | Nebraska, USA |
| *Sclerotinia sclerotiorum* (Lib.) De Bary | LMK44 | 1837.P | | *Lactuca sativa* | Ontario, Canada |
| *Sclerotinia sclerotiorum* | LMK57 | 1839.P | *Ranunculus ficaria* | | Akershus, Norway |
| *Sclerotinia sclerotiorum* | LMK754 | 2335.P | *Ranunculus ficaria* | | Telemark, Norway |
| *Sclerotinia* species 1 | LMK745 | 1707.2 | *Taraxicum* | | Trondelag, Norway |
| *Sclerotinia trifoliorum* Erikss. | LMK36 | 1887.P / ATCC34327 | | *Trifolium repens* | Tasmania |
| *Sclerotinia trifoliorum* | LMK47 | 1888.P | *Medicago sativa* | | Virginia, USA |
| *Sclerotium cepivorum* Berk. | LMK1 | 1878.S | | *Allium cepa* | Netherlands |
| *Sclerotium cepivorum* | LMK71 | 1889.S | *Allium cepa* (soil) | | New Jersey, USA |
| *Verpatinia calthicola* Whetzel | LMK756 | 2442.P | *Iris pseudacorus* | | Telemark, Norway |
| *Verpatinia spiraeicola* Dennis | LMK758 | 2445.P | *Filipendula ulmaria* | | Telemark, Norway |

^a^ LMK numbered strains refer to culture numbers in L.M. Kohn’s culture collection in the University of Toronto, Canada.

^b^ Unless otherwise stated, the isolate codes refer to culture numbers in the University of Oslo, Norway. Other codes are: ATCC number = culture number in American type culture collection; CUP number = culture number in Plant Pathology Herbarium, Cornell University, NY

^c^ The annotated genome for *B. cinerea* strain B05.10 is publically available through the Broad Institute, Cambridge, MA (http://www.broad.mit.edu/annotation/genome/botrytis_cinerea/Home.html).

^d^ haploid strain derived from SAS56 isolate – source: *Vitis* (ascospore line), origin: Italy

^e^ The annotated genome for *S. sclerotiorum* strain 1980 (ATCC18683) is publically available through the Broad Institute, Cambridge, MA (http://www.broad.mit.edu/annotation/genome/sclerotinia_sclerotiorum/Home.html)
