# Supplementary material for: Evidence for a Common Toolbox Based on Necrotrophy in a Fungal Lineage Spanning Necrotrophs, Biotrophs, Endophytes, Host Generalists and Specialists
Source: PLoS One. 2012 Jan 11;7(1):e29943. doi: 10.1371/journal.pone.0029943 (PMC3256194; doi:10.1371/journal.pone.0029943)
Supplement: Table S4 — Primer sets for amplification of housekeeping and pathogenicity-related genes in the Sclerotiniaceae. (DOC) [file pone.0029943.s012.doc]

Table S4. Primer sets for amplification of housekeeping and pathogenicity-related genes in the Sclerotiniaceae.

| **Locus** | **Definition** | **Primer name** | **Primer DNA sequence (5’-3’)** | **Optimal annealing temp. (ºC)** |
| --- | --- | --- | --- | --- |
| *cal* | Calmodulin | CAL-228F | GAGTTCAAGGAGGCCTTCTCCC | 55 |
|  |  | CAL-737R | CATCTTTCTGGCCATCATGG |  |
| *g3pdh* | Glyceraldehyde-3-phosphate | G3PDH-Fbis | GCTGTCAACGACCCTTTCAT | 58 |
|  |  | G3PDH-Rbis | ACCAGGAAACCAACTTGACG |  |
| *hsp60* | Heat shock protein 60 | HSP60for-deg | CAACAATTGAGATTYGCCCAYAAG | 53 |
|  |  | HSP60rev-deg | GATRGATCCAGTGGTACCGAGCAT |  |
| *acp1* | Acid protease 1 | ACPF-deg | GCWGCYCCAGGTACKGC | 50 |
|  |  | ACPR-deg | CGAGRARYTRCCYTCCT |  |
| *asps* | Aspartyl protease | ASPSaF-deg | GGTGCYGGTACMAGAG | 53 |
|  |  | ASPSaR-deg | GGCTTRACRGTGTTSAG |  |
|  |  | ASPSbF-deg | ACATYGGWGGDGYVACTGT | 55 |
|  |  | ASPSbR-deg | TTRAACATRATGTCRCCGTA |  |
| *oah* | Oxaloacetate acetylhydrolase | OAHaF | CGAGTTGCCCTTCAAGTT | 50 |
|  |  | OAHaR-deg | AGATGYCCACAACGCTTGTT |  |
|  |  | OAHbF-deg | CCTCTCATYRTTGACAAAGC | 50 |
|  |  | OAHbR-deg | CCACCAGCCGYAGTAT |  |
| *pac1* | Zinc finger transcription factor | PAC1aF-deg | ATGCAYCCTGGTTCTGC | 58 |
|  |  | PAC1aR | TGGTGCAGTTGCTGAATG |  |
|  |  | PAC1bF | TCTGGTACACCAGCTTTAACAC | 55 |
|  |  | PAC1bR-deg | CTCATCATCATCATCCYGTTTTCA |  |
| *pg1* | Polygalacturonase 1 | PG1aF-deg | TGGCAATGGRGWGGMA | 50 |
|  |  | PG1aR-deg | GCVYCHGMCCADACYTT |  |
|  |  | PG1bF-deg | TCYGTDGGVAGYTTAGG | 60 |
|  |  | PG1bR-deg | CAYTACTRCAYACCAAAGT |  |
| *pg3* | Polygalacturonase 3 | PG3aF-deg | CCCTRTCCATCCCAGTATGC | 58 |
|  |  | PG3aR-deg | TGCYTGGCGACAACCCAGA |  |
|  |  | PG3bF-deg | ACGTCCACCATYGATCTTCAAAA | 58 |
|  |  | PG3bR-deg | GTCTTTCCACCTCCAGARATGCT |  |
| *pg5* | Polygalacturonase 5 | PG5aF-deg | TTRMYAAGCGRGCAAC | 55 |
|  |  | PG5aR-deg | ATAGCMACRCARTCRTCTT |  |
|  |  | PG5bF-deg | TGGGATGGAVAWGG | 55 |
|  |  | PG5bR-deg | ATACCGTAGTCGGTRATRC |  |
| *pg6* | Polygalacturonase 6 | PG6aF-deg | GGGCTYTTAGCCCTCACYTT | 55 |
|  |  | PG6aR-deg | TTTCCACCWACAGATCCGATG |  |
|  |  | PG6bF-deg | CAATCAGGATGACTGCGTMGC | 55 |
|  |  | PG6bR-deg | CAGCCRGTTGMTGGRTAGTTACA |  |
