# Supplementary material for: Evidence for a Common Toolbox Based on Necrotrophy in a Fungal Lineage Spanning Necrotrophs, Biotrophs, Endophytes, Host Generalists and Specialists
Source: PLoS One. 2012 Jan 11;7(1):e29943. doi: 10.1371/journal.pone.0029943 (PMC3256194; doi:10.1371/journal.pone.0029943)
Supplement: Table S5 — Supporting information on loci used in phylogenetic analyses of housekeeping and pathogenicity-related loci. (DOC) [file pone.0029943.s013.doc]

**Table S5. Supporting information on loci used in phylogenetic analyses of housekeeping and pathogenicity-related loci**.

| **Locus** | **Sequence length (bp)** | **Number of parsimony-informative sites** | **Model of DNA sequence evolution** ^a^ | **Base frequencies** | **Rate matrix** | **Shape parameter for gamma distribution** | **Proportion of invariant sites** |
| --- | --- | --- | --- | --- | --- | --- | --- |
| *cal* | 497 | 296 | TrN+I+G | 0.2509, 0.2795,  0.2102, 0.2594 | 1.0000, 4.6610, 1.0000,  1.0000, 4.5560, 1.0000 | 3.3559 | 0.2526 |
| *g3pdh* | 773 | 258 | GTR+I+G | 0.2254, 0.3098,  0.2348, 0.2300 | 1.0193, 1.8308, 1.4540,  0.6057, 6.6433, 1.0000 | 0.7305 | 0.3227 |
| *hsp60* | 924 | 372 | GTR+I+G | 0.2477, 0.2868, 0.2359, 0.2296 | 1.6342, 4.6874, 1.3592,  0.6545, 10.6397, 1.0000 | 1.2447 | 0.4432 |
| *acp1* | 666 | 120 | GTR+G | 0.2034, 0.3098,  0.2409, 0.2459 | 6.4683, 16.2086, 14.7476,  4.2363, 27.3118, 1.0000 | 0.4884 | 0 |
| *asps* | 1207 | 546 | GTR+I+G | 0.2495, 0.3208,  0.2119, 0.2178 | 1.4623, 3.6171, 2.0587,  1.2785, 6.1529, 1.0000 | 1.7816 | 0.3465 |
| *oah* | 889 | 318 | SYM+I+G | 0.2500, 0.2500,  0.2500, 0.2500 | 1.8102, 3.8039, 1.3102,  0.6711, 6.0747, 1.0000 | 0.4567 | 1.9715 |
| *pac1* | 1337 | 406 | GTR+G | 0.2827, 0.2447, 0.1964, 0.2762 | 2.1964, 5.6973, 1.6225, 1.1248, 6.8885, 1.0000 | 0.4298 | 0 |
| *pg1* | 1093 | 383 | GTR+ G | 0.2939, 0.2007,  0.2182, 02872 | 1.5025, 2.9957, 0.8320,  0.7013, 5.0201, 1.0000 | 0.5194 | 0 |
| *pg3* | 1449 | 435 | TrN+G | 0.2514, 0.2981, 0.2057, 0.2448 | 1.0000, 3.2045, 1.0000,  1.0000, 4.2913, 1.0000 | 0.4997 | 0 |
| *pg5* | 1119 | 432 | GTR+I+G | 0.2737, 0.2416,  0.2099, 0.2748 | 1.9003, 4.6263, 1.1184,  1.4684, 7.2334, 1.0000 | 2.4168 | 0.3628 |
| *pg6* | 1091 | 367 | GTR+G | 0.2859, 0.2335, 0.2101, 0.2705 | 2.5278, 7.2901, 1.5685,  2.5575, 10.9278, 1.0000 | 0.4733 | 0 |

^a^ The models of DNA sequence evolution (GTR - general time reversible model, TrN - Tamura-Nei model and SYM model) are presented with I – invariant sites, and/or G – gamma-distributed sites.
