# Supplementary material for: Evidence for a Common Toolbox Based on Necrotrophy in a Fungal Lineage Spanning Necrotrophs, Biotrophs, Endophytes, Host Generalists and Specialists
Source: PLoS One. 2012 Jan 11;7(1):e29943. doi: 10.1371/journal.pone.0029943 (PMC3256194; doi:10.1371/journal.pone.0029943)
Supplement: Table S6 — Primer/probe sets for quantitative PCR of 2 pathogenicity-related loci (oah and pac1) and a reference gene (actin). (DOC) [file pone.0029943.s014.doc]

| ***oah* locus:** | | | | | | | |
| --- | --- | --- | --- | --- | --- | --- | --- |
| **Strain** | **Primer F** | **Sequence (5’-3’)** | | **Primer R** | **Sequence (5’-3’)** | **Probe** | **Sequence (5’-3’)** |
| B05.10 | oah_859Fa | CCAATCATCACCACCAAG | | oah_928Ra | CGAGAGCAGCAAAAGAGA | oah_902a | ATACGGAATCCCATCTCTTGTGC |
| 1980 | oah_859Fb | CCTATCATCACCACCAAG | | oah_928Rd | CGAGAGCAGCGAAGGAGA | oah_902a | ATACGGAATCCCATCTCTTGTGC |
| 735 | oah_859Ff | CCTATAATTACTACCGCA | | oah_928Re | CGAGAGCAGCGAAAGAAA | oah_902a | ATACGGAATCCCATCTCTTGTGC |
| 746 | oah_859Fe | CCTATAATTACCACCGCA | | oah_928Rb | CGAGAGCAGCAAAAGAAA | oah_902a | ATACGGAATCCCATCTCTTGTGC |
| 743 | oah_859Fc | CCCATCATCACCACCAAG | | oah_928Rc | CGAGAGCAGCAAAGGAGA | oah_902a | ATACGGAATCCCATCTCTTGTGC |
| 76 | oah_859Fa | CCAATCATCACCACCAAG | | oah_928Ra | CGAGAGCAGCAAAAGAGA | oah_902a | ATACGGAATCCCATCTCTTGTGC |
| 125 | oah_859Fi | CCCATTATCACCACCAAA | | oah_928Rh | CGAGAGCCGCAAATGAGA | oah_902b | ATGCGGAATCCCATCTCTTGTGC |
| 733 | oah_859Fh | CCTGTCATCACGACCAAG | | oah_928Rg | CGAGAGCGGCAAATGAGA | oah_902c | ATGCGGAATCCCATCTCTTGAGC |
| 738 | oah_859Fg | CCTGTGATCACTACCGCA | | oah_928Rf | CAAGAGCAGCAAAAGAAA | oah_902b | ATGCGGAATCCCATCTCTTGTGC |
| ***pac1* locus:** | | | | | | | |
| **Strain** | **Primer F** | **Sequence (5’-3’)** | **Primer R** | | **Sequence (5’-3’)** | **Probe** | **Sequence (5’-3’)** |
| B05.10 | pac1_609Fa | AATGGCACCACATGTTTC | pac1_692Ra | | GGTGTACCAGATGAAACT | pac1_627a | CTTCTTACGCCTCAGCACCAA |
| 1980 | pac1_609Fc | AATGGCGCCACATGTTTC | pac1_692Rc | | GGTGTACCAGAAGAAACT | pac1_627c | CTTCTTACGCTTCAGCACCAA |
| 735 | pac1_609Fa | AATGGCACCACATGTTTC | pac1_692Ra | | GGTGTACCAGATGAAACT | pac1_627c | CTTCTTACGCTTCAGCACCAA |
| 746 | pac1_609Fa | AATGGCACCACATGTTTC | pac1_692Ra | | GGTGTACCAGATGAAACT | pac1_627c | CTTCTTACGCTTCAGCACCAA |
| 743 | pac1_609Fb | AATGCCACCGCATGTTTC | pac1_692Rb | | GGTGTACCAGATGAAACC | pac1_627b | CTTCTTATGCTTCGGCACCAA |
| 76 | pac1_609Fa | AATGGCACCACATGTTTC | pac1_692Ra | | GGTGTACCAGATGAAACT | pac1_627a | CTTCTTACGCCTCAGCACCAA |
| 125 | pac1_609Fb | AATGCCACCGCATGTTTC | pac1_692Rb | | GGTGTACCAGATGAAACC | pac1_627d | CTTCCTATGCGTCAGCACCAA |
| 733 | pac1_609Fd | AATGCCACCACATGTTTC | pac1_692Rb | | GGTGTACCAGATGAAACC | pac1_627b | CTTCTTATGCTTCGGCACCAA |
| 738 | pac1_609Fa | AATGGCACCACATGTTTC | pac1_692Ra | | GGTGTACCAGATGAAACT | pac1_627c | CTTCTTACGCTTCAGCACCAA |
| ***actin* locus:** | | | | | | | |
| **Strain** | **Primer F** | **Sequence (5’-3’)** | | **Primer R** | **Sequence (5’-3’)** | **Probe** | **Sequence (5’-3’)** |
| B05.10 | act_88Fa | GTTTTCCCTTCCATTGTC | | act_174Ra | CGCTTCATCTCCAACATA | act_149a | TTGACCCATACCAATCATAATACCA |
| 1980 | act_88Fa | GTTTTCCCTTCCATTGTC | | act_174Ra | CGCTTCATCTCCAACATA | act_149a | TTGACCCATACCAATCATAATACCA |
| 735 | act_88Fb | GTTTTCCCTTCTATTGTC | | act_174Rb | CGCTTCATCTCCGACATA | act_149b | TTGACCCATACCAATCATGATACCA |
| 746 | act_88Fb | GTTTTCCCTTCTATTGTC | | act_174Ra | CGCTTCATCTCCAACATA | act_149b | TTGACCCATACCAATCATGATACCA |
| 743 | act_88Fa | GTTTTCCCTTCCATTGTC | | act_174Ra | CGCTTCATCTCCAACATA | act_149a | TTGACCCATACCAATCATAATACCA |
| 76 | act_88Fa | GTTTTCCCTTCCATTGTC | | act_174Ra | CGCTTCATCTCCAACATA | act_149a | TTGACCCATACCAATCATAATACCA |
| 125 | act_88Fa | GTTTTCCCTTCCATTGTC | | act_174Rc | TGCTTCATCTCCAACATA | act_149a | TTGACCCATACCAATCATAATACCA |
| 733 | act_88Fa | GTTTTCCCTTCCATTGTC | | act_174Rc | TGCTTCATCTCCAACATA | act_149c | TTGACCCATACCAATCATAATACCG |
| 738 | act_88Fa | GTTTTCCCTTCCATTGTC | | act_174Rc | TGCTTCATCTCCAACATA | act_149a | TTGACCCATACCAATCATAATACCA |

**Table S6. Primer/probe sets for quantitative PCR of 2 pathogenicity-related loci (*oah* and *pac1*) and the reference gene (*actin*).**
